# Prediction of moisture-induced cracks in wooden cross sections using finite element simulations

**DOI:** 10.1007/s00226-023-01469-3

**Published:** 2023-04-27

**Authors:** Florian Brandstätter, Maximilian Autengruber, Markus Lukacevic, Josef Füssl

**Affiliations:** TU Wien, Institute for Mechanics of Materials and Structures, Karlsplatz 13, 1040 Vienna, Austria

## Abstract

Wood absorbs and desorbs moisture due to its hygroscopic behavior, leading to moisture gradients in timber elements as well as swelling and shrinkage. These processes are constrained due to the orthotropic material properties of wood, leading to moisture-induced stresses, which can cause crack initiation and propagation. A significant amount of the damage in timber constructions indoors can be related to changes of the moisture content (MC). However, more information is needed about the correlation between moisture changes or gradients and specific damage characteristics, like crack depths. Thus, based on numerical simulations, the crack depth development within two solid timber and one glued laminated timber (GLT) cross section over time for different relative humidity (RH) reductions and initial MCs is analyzed. For this purpose, a multi-Fickian transport model is used to determine moisture fields, which are then used as loads in a subsequent stress simulation, where linear elastic material behavior is considered. An extended finite element approach, supported by a multisurface failure criterion defining the failure behavior, allows for the simulation of moisture-induced discrete cracking. Based on simulation results, correlations between potential maximum crack depths and moisture gradients in indoor climate conditions are derived, which enables the prediction of crack depths in wood. Finally, it is shown that the initial MC level significantly influences the maximum crack depth that can be expected.

**Supplementary Information:**

The online version contains supplementary material available at 10.1007/s00226-023-01469-3.

## Introduction

Wood is a building material that is significantly affected by moisture. Many material parameters, e.g., density, strength and stiffness, depend on the moisture content (MC) as the cell walls absorb and desorb moisture due to their hygroscopicity (Siau 1984). In wood, moisture occurs as bound water in the wooden cells as well as water vapor and free water in the lumen, where free water is only present if the bound water concentration reaches the fiber saturation point (FSP). Variations in MC result in volume changes as well as moisture gradients from the surface to the interior of wooden members (Svensson et al. 2011). Due to orthotropic material behavior of wood, swelling and shrinkage processes caused by moisture gradients are constrained, leading to moisture-induced stresses (Fragiacomo et al. 2011), which differ in the directions characteristic for wood (longitudinal, radial and tangential). An increase of the stress level can lead to initiation and propagation of cracks causing a reduction in the load-bearing capacity.

Moisture transport in wood below the FSP can be described by the diffusion processes of bound water in the cells and water vapor in the lumen, coupled by sorption (Frandsen et al. 2007a). Possible approaches to describe these mechanisms are single-Fickian or multi-Fickian models. While for the single-Fickian approach, one governing equation describes moisture flow (Florisson et al. 2020; Frandsen et al. 2007a), for the multi-Fickian one, two governing equations (one for bound water and one for water vapor) define moisture transport (Frandsen et al. 2007a; Krabbenhøft and Damkilde 2004). The single-Fickian model might be acceptable for problems below 65 % relative humidity (RH) (Frandsen et al. 2007a; Konopka and Kaliske 2018) if small moisture gradients occur (thin bodies, small or slow RH changes; Konopka and Kaliske , 2018) and errors are tolerable to certain degrees, whereas the multi-Fickian approach enables the examination of more sophisticated issues, such as the determination of realistic moisture states for timber bridges (Fortino et al. 2013), the simulation of the hygro-thermal behavior of coated components of a timber bridge (Fortino et al. 2019), or the implementation of free water transport (Autengruber et al. 2020).

Various works study the effect of moisture on wood, which can influence cracking behavior. Svensson et al. (2011) examined the effects of harmonic RH cycles with variations in amplitude, period, and mean value of RH on the moisture gradient, the so-called penetration depth, and the MC amplitude on the surface of a timber member. It was shown that the period immensely affects the penetration depth, which is the distance from the surface to the point where maximum MC fluctuations of the MC envelope are 0.5 %, and the cycle’s amplitude significantly influences the moisture gradient. As mentioned before, moisture gradients are related to stresses, which was investigated in Fragiacomo et al. (2011). There, it was shown that Northern European climates result in larger moisture gradients, and consequently, in higher stresses compared to Southern European climates. Other scientific works emphasize the significance of moisture-induced cracking as well, such as in Dietsch (2017). There, the effects of moisture-induced stresses, which were caused by swelling and shrinkage restriction due to reinforcement with threaded screws or steel rods positioned perpendicular to the grain on glued laminated timber beams, were examined. Reducing the MC by 1 % already decreased the benefits of reinforcing timber members. Besides, a moisture difference of 3 % already induced sufficient stress to initiate large cracks around the fully threaded screws or steel rods. Moreover, Huč et al. (2020) determined that moisture-induced stresses caused by drying in glued laminated timber were greater than the limits of the tensile strength perpendicular to the grain of spruce wood. Further investigations of different climates on moisture-induced stresses were conducted by Angst and Malo (2012), where glulam cross sections were exposed to wetting and drying cycles during the experiments. It was shown that the determined internal stresses perpendicular to the grain can exceed the strength of wood, leading to cracking.

Crack initiation and propagation can be described by fracture mechanics. For wood, several research works examine this issue to improve the realistic modeling of wood fracture by, e.g., introducing a new fracture mechanics theory based on a orthotropic–isotropic transformation of the Airy stress function (van der Put 2007a) or making suggestions for improvement of the softening curve and pointing out previous errors regarding fracture energy (van der Put 2007b). Other works focus on improved modeling of applications, such as Larsson et al. (2016), analyzing the fracture behavior of bond lines of glued wood-to-steel plate joints, and Serrano (2004), studying shear-strength estimation of bonds between wood and adhesive used.

Crack formation can be implemented in finite element simulations using the so-called extended finite element method (XFEM), based on the work of Melenk and Babuška (1996) and Belytschko and Black (1999). Additional degrees of freedom and special displacement functions enable the consideration of discontinuities, such as cracks, within the now enriched finite elements, independent of the underlying mesh (Konopka et al. 2017). XFEM was used to examine cracking in wood in various works, investigating bending of glued laminated timber (GLT) beams (Qiu et al. 2014; Vida et al. 2022), modeling brittle failure of wooden structures (Gebhardt and Kaliske 2020), finding optimal parameter values for numerical modeling of fracture using experiments as a basis (Ostapska and Malo 2021) or moisture-induced crack initiation and propagation of circular cross sections made of Anhui fir (Chen et al. 2019).

Damage in wood indoors can be caused by numerous conditions. Dietsch et al. (2015) showed that about 50 % of the damages are related to low and high MC as well as MC changes, where approximately half of all damages are caused by cracks. The most significant influence on the moisture content is exerted from the climate surrounding the construction and its variations, which can differ significantly whether outdoor or indoor conditions are investigated, as the following example shows: While outdoor RH can exceed 40 % several times in a period of days or even weeks, indoor RH can remain below 40 % during the same time (Bertolin et al. 2015; Ferdyn-Grygierek 2014; Nguyen et al. 2014), providing a more crack-prone environment.

As moisture-induced crack formation can impair the load-bearing capacity as well as the durability of a wooden component, it is essential to know under which conditions the most severe moisture variations occur. The greatest risk of comparatively intensive moisture changes appears during the first winter or from the beginning of erection until commissioning (Dietsch et al. 2015). During winter the indoor RH can drop to an average of 30.0 % (Ferdyn-Grygierek 2014; Hameury and Lundström 2004; Nguyen et al. 2014; Log 2017) for about two to four weeks resulting in an equilibrium MC of 8.2 %. In rare cases (Alsmo and Alsmo 2014), even an average indoor RH of 25.0 % (7.2 % MC) over 30 days (d) and 20.0 % (6.3 % MC) over 22.5 d are possible, respectively. However, most wooden timber members show an initial MC between 10 % and 12 % after production (Dietsch 2017), and according to the standard DIN EN 14081-1 (2019), a measurement error of $\pm$ 3 % MC during production is tolerable. This difference in MC shows the possible damage potential of moisture-induced cracking, which can be additionally influenced by air conditioning. Ferdyn-Grygierek (2014) investigated environmental parameters of a museum, which was equipped with air conditioning, for one year. The results show that an average RH of 30.0 % was recorded for at least 15 d in January. Hameury and Lundström (2004) examined the indoor climate of wooden residential buildings revealing an average RH of 30 % over at least 22.5 d in January. Nguyen et al. (2014) analyzed the correlation between indoor climate and outdoor conditions based on measurements in 16 homes in the eastern USA, where the buildings were equipped either with or without air conditioning, showing that in winter the RH decreased to an average of 30 % for at least 30 d. Log (2017) studied whether indoor RH is suitable as fire risk indicator. The measurements revealed that in extreme cases, with a ventilation system, the RH was below 30 % for at least 30 d. However, without air conditioning, an average RH of 30 % could occur as well, but only between one and three weeks, with the RH never decreasing below 30 % in some buildings. Alsmo and Alsmo (2014) analyzed the relationship between RH and respiratory infections, where measurements showed that the RH can decrease to an average of 25 % over 30 d in case of natural ventilation in January. However, if mechanical ventilation is installed, the RH can drop to an average of 25 % over 60 d and to an average of 20 % over 22.5 d, respectively, during winter. The given literature indicates that air conditioning is not necessary to induce a low RH but influences it significantly.

Several works investigated moisture-induced stresses in wood exposed to outdoor climate conditions (Autengruber et al. 2021a; Fortino et al. 2013, 2019; Fragiacomo et al. 2011), but less is known about moisture-induced stresses in indoor climate conditions (Dietsch et al. 2015). In addition, no quantitative relation between moisture gradients and crack depth exists.

In this work, we aim to relate and predict moisture gradients and crack depths in wood cross sections exposed to indoor climate conditions by investigating various drying scenarios simulated numerically. Two solid timber (ST) and one glued laminated timber cross sections are investigated under varying initial MC and different drying loads in "Cracking behavior of different cross sections"section. Therefore, while keeping the temperature constant, the RH is decreased in a single step when the simulation is initialized, which changes the moisture field of the cross sections resulting in shrinkage strains. To determine whether the stresses induced in the process lead to cracking, a multisurface failure criterion (Lukacevic et al. 2017; Lukacevic and Füssl 2016; Lukacevic et al. 2015; Li et al. 2018) is used. XFEM is utilized to simulate the initiation and the propagation of cracks. With the results of the drying scenarios, the relation between crack depth and moisture gradient is analyzed in "Relation of crack depth and moisture gradient" section. In "Influence of reducing relative humidity linearly over time on crack development" section, the influence of linearly reducing RH over time on the cracking behavior is examined. The results from Cracking behavior of different cross sections" section enable the crack depth prediction in indoor climate conditions, which is simplified in the Sect. "Simplified crack depth determination for indoor climate conditions" . It is assumed that wooden beams are installed under ideal conditions, where the MC in timber cross sections is kept constant during production until the beginning of operation.

## Materials and methods

**Fig. 1:**
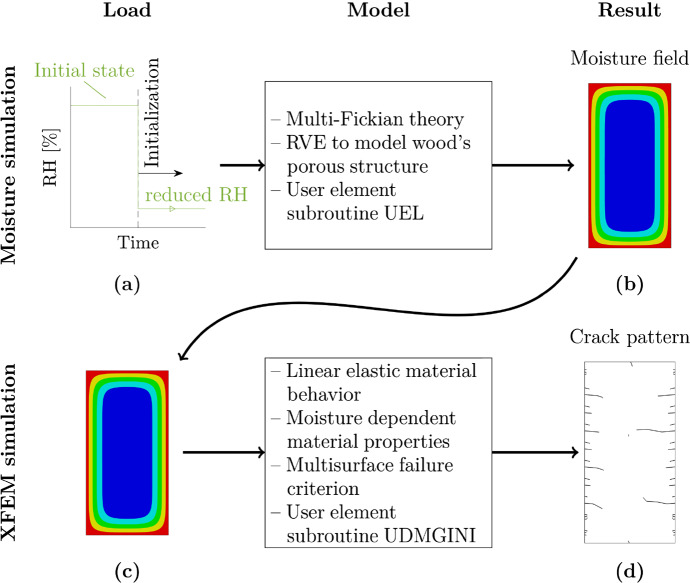
Schematic description of the cracking progress. Based on relative humidity (RH) reductions, for the three cross sections, moisture fields were determined in a first simulation. In a subsequent XFEM simulation, using the moisture fields as loading, stresses were determined based on linear elastic material behavior. For each increment in each integration point, a multisurface failure criterion was evaluated. If the criterion was violated, cracks initiate or propagate

To relate and predict moisture gradients and crack depths, a model describing the moisture transport mechanisms and a model for stress simulation as well as crack initiation and propagation are introduced. Figure 1 shows the different components of the models used. The initial MC is derived from the initial RH, which is reduced to various levels. Based on these RH reductions (drying scenarios), for the three cross sections, moisture fields are determined, where for the moisture transport, a multi-Fickian transport model is used. A representative volume element (RVE) is utilized to model the porous structure of wood and consider the influence of the water states (bound water and water vapor) on moisture transport correspondingly. Using the moisture field as loading, in a subsequent XFEM simulation, stresses caused by constrained swelling and shrinkage are determined based on linear elastic material behavior. However, effects such as viscoelasticity, mechano-sorption and plastification are not considered during the calculations. In this manuscript, moisture-induced stresses are only based on linear elastic material behavior. For the strength of wood, a multisurface failure criterion, introduced by Lukacevic et al. (2017), is used. During the XFEM simulation, in each integration point, the criterion is evaluated. If the stresses exceed the strength limit, cracks initiate or propagate.

For the simulations, the finite element software *Abaqus* (Abaqus Documentation 2014) is used. The cross sections were discretized using brick elements with linear interpolation functions (C3D8). The systems of equations are solved with the modified Newton method. To be able to consider different pith locations, the material is defined as cylindrical-orthotropic in each integration point under consideration of the local material orientation.

### Mathematical model for moisture transport in wood

For the moisture transport model the multi-Fickian theory of Eitelberger et al. (2011), Fortino et al. (2013, 2019), Frandsen et al. (2007a), Konopka and Kaliske (2018) and Krabbenhøft and Damkilde (2004) was used, which describes moisture flow based on two coupled diffusion processes: The transport of water vapor $c_v$ in the lumen and the transport of bound water $c_b$ within the cells. Both are coupled by the sorption rate $\dot{c}_{bv}$ under consideration of a hysteresis effect. It is assumed that the MC of the cross section never reaches the FSP, preventing the occurrence of free water in the wooden cells.

The diffusion processes as well as the energy conservation, which is also required, can be described by differential equations given in  Autengruber et al. (2021a):

*Conservation of bound vapor concentration:*

1 $$\begin{aligned} \begin{aligned} \frac{\partial c_b}{\partial t} = \frac{\partial }{\partial \varvec{x}} \cdot \varvec{D_b} \cdot \frac{\partial c_b}{\partial \varvec{x}} + \dot{c}_{bv}\\ \end{aligned} \end{aligned}$$

*Conservation of water vapor concentration:*

2 $$\begin{aligned} \begin{aligned} \frac{\partial c_v }{\partial t} f_{lum} =&\frac{\partial }{\partial \varvec{x}} \cdot \varvec{D_v} \cdot \frac{\partial c_v}{\partial \varvec{x}} f_{lum} - \dot{c}_{bv}\\ \end{aligned} \end{aligned}$$

*Conservation of energy:*

3 $$\begin{aligned} \begin{aligned} \frac{\partial (\rho \, h)}{\partial t} =&\frac{\partial }{\partial \varvec{x}} \cdot \varvec{K} \cdot \frac{\partial T}{\partial \varvec{x}} + \frac{\partial }{\partial \varvec{x}} \cdot \varvec{D_b} \cdot \frac{\partial c_b}{\partial \varvec{x}} \overline{h}_b + \frac{\partial }{\partial \varvec{x}} \cdot \varvec{D_v} \cdot \frac{\partial c_v}{\partial \varvec{x}} h_v f_{lum} \\&+ \dot{c}_{bv} \, (h_v - h_b) \end{aligned} \end{aligned}$$

The left-hand sides of Equations (1) to (3) describe the rate of change of the concentrations $c_b$ and $c_v$ as well as of the energy ($\rho \, h$). While the transport tensors of bound water $\varvec{D_{b}}$ and water vapor $\varvec{D_{v}}$ account for the diffusion processes, the conduction tensor $\varvec{K}$ denotes the thermal conduction. To account for the corresponding volume, $c_v$ is related to the volume proportion of the lumen $f_{lum}$ and $c_b$ to the whole volume of the representative volume element (RVE).

In Equation (3), $\overline{h}_b$ and $h_b$ describe the average and specific enthalpy of bound water, respectively, and $h_v$ is the enthalpy of water vapor. The change of the internal energy over time $\Delta t$ is defined by:

4 $$\begin{aligned} \begin{aligned} (\rho \, h)_{t+\Delta t} - (\rho \, h)_{t} =&\, \rho _d \, c_{p_s} \, (T_{t+\Delta t} - T_{t}) + c_{b_{t+\Delta t}} \, \overline{h}_{b_{t+\Delta t}} - c_{b_{t}} \, \overline{h}_{b_{t}} \\ & + c_{v_{t+\Delta t}} \, h_{v_{t+\Delta t}} \, f_{lum_{t+\Delta t}} -c_{v_{t}} \, h_{v_{t}} \, f_{lum_{t}} \end{aligned} \end{aligned}$$

In Tables 1 and 2, in Online Resource 1, all constitutive equations and the material parameters are given.

Equations (1) to (3) were implemented in the moisture transport model with the user element subroutine UEL (Abaqus Documentation 2014).

As presented in Frandsen et al. (2007b), the occurring interaction between bound water and water vapor is described by the sorption rate. Detailed information about the interaction between bound water and water vapor can be obtained from Section 2 in Online Resource 1. Since the temperature was kept constant during the simulations, an isothermal hysteresis is assumed for this process.

#### Initial conditions for stress determination

The initial temperature $T_{ini}$ and the initial water vapor concentration $c_{v,ini}$, depending on the initial relative humidity $\textrm{RH}_{ini}$, form the basis of the calculations, as they describe the initial bound water concentration $c_{b,ini}$. $c_{b,ini}$ and $c_{v,ini}$ are in equilibrium, as it is assumed that the wooden element was kiln dried from green wood conditions to the required moisture content level.

#### Boundary conditions

Boundary conditions define the fluxes through the exchange surfaces considering the surrounding climate. The water vapor flux $\phi _v$ is described as

5 $$\begin{aligned} \begin{aligned} \phi _v = k_{{c}_v} (c_v - c_{v,0}&)f_{lum} \\ k_{{c}_v} = \frac{Sh}{L} \, D_{air} \end{aligned} \end{aligned}$$

with the film boundary coefficient $k_{{c}_v}$. It considers convection and air flow depending on the air speed and the potential coatings. $c_{v,0}$ is the water vapor concentration of the surrounding climate. The determination of the film boundary coefficient is based on Eitelberger et al. (2011), where the Sherwood number Sh is 1 and L is 0.035 m. $D_{air}$ is the diffusion coefficient of water vapor in air (see Table 1 in Online Resource 1) with ${\varvec{\xi }}$ of 1.

In addition to the water vapor flux, the flux of energy $\phi _T$ also has to be considered:

6 $$\begin{aligned} \phi _T = k_T (T - T_0) + k_{{c}_v} (c_v - c_{v,0}) f_{lum} h_v \end{aligned}$$

with $k_T$ as the heat transfer coefficient and $T_0$ as the temperature of distant air. The temperature is kept constant at 293.15 K.

#### Drying scenarios

**Fig. 2:**
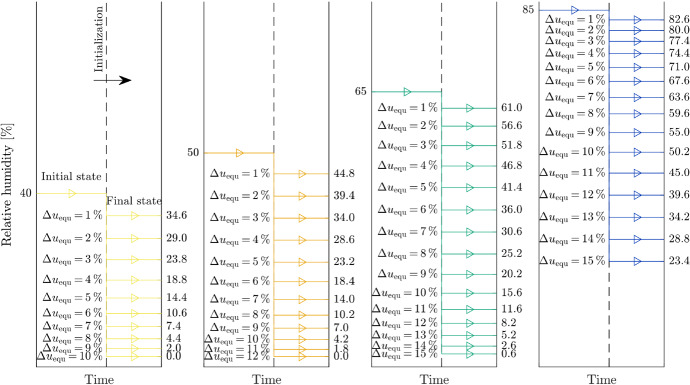
Overview of the single-step relative humidity (RH) reductions at the boundary, leading to equilibrium MC differences $\Delta u_{\textrm{equ}}$ of 1 % up to 15 % from four initial RH levels (40 %, 50 %, 65 % and 85 %) determined with the desorption isotherm (Frandsen et al. 2007b)

The drying scenarios are defined with different initial MCs and are characterized by the following steps: At the beginning, the initial moisture field is uniform across the whole cross section depending on the initial RH and considering the desorption isotherm at 293.15 K (initial state). Then, the RH is reduced in a single step to a certain level (final state), which is held constant for 30 days, resulting in non-uniform moisture distributions. The reduction of the RH is defined in such a way that a difference in equilibrium MC $\left( \Delta u_{\textrm{equ}} \right)$ of 1 % up to 15 % is achieved. To study the effect of the nonlinear definition of the used desorption isotherm, the differences in MCs are combined with four different initial MC levels (10.0 %, 11.9 %, 15.3 % and 22.0 %) corresponding to RH levels of 40.0 %, 50.0 %, 65.0 % and 85.0 %, respectively. This results in 52 configurations, shown in Fig. 2. The definition of the initial levels is based on the RH, as the RH is easier to measure at the installation location than the equilibrium MC. The initial RH values are based on the limits of the service classes in EC 5 (ÖNORM B 1995-1-1 2019), since they are used to consider the effects of moisture and temperature on deformation as well as strength and, thus, the load-bearing capacity of timber structures. While the initial MC levels 10.0 % and 11.9 % correspond to appropriate transport and storage conditions, the initial MC levels 15.3 % and 22.0 % relate to unfavorable situations, where, e.g., timber components are exposed to outdoor climate during transport and storage without sufficient weather protection.

### Model for fracture in wood

Following the moisture simulations, XFEM simulations are performed, using the moisture simulation results as loading, to determine moisture-induced stresses, caused by constrained shrinkage. For the XFEM simulations, moisture-dependent material parameters are introduced and the strength of wood is defined by a multisurface failure criterion described in Lukacevic et al. (2017), which is evaluated in each integration point for each increment throughout the XFEM simulation. If the stresses exceed the failure criterion, crack initiation or propagation in an element occurs.

**Fig. 3:**
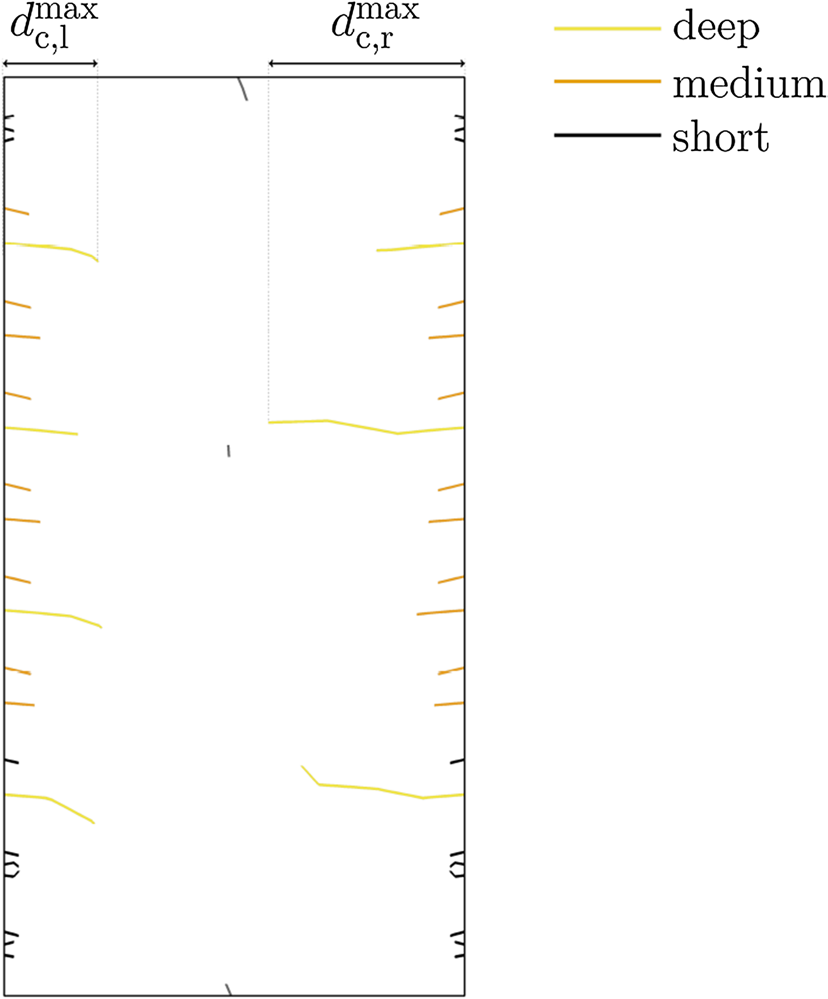
Exemplary crack pattern of the cross section GLT 20 $\times$ 40. Depending on their depth, the horizontal cracks can be qualitatively divided into three groups (deep, medium and short). Additionally, the sum of the deepest cracks at both the left and the right cross section edge ($d_{\textrm{c,l}}^{\textrm{max}}$ and $d_{\textrm{c,r}}^{\textrm{max}}$) is defined as the maximum total crack depth $d_{\textrm{c}}^{\textrm{max}}$

To describe the crack pattern evolution, the development of the crack depth is investigated. Therefore, in a subsequent calculation, the sizes of the cracks are analyzed. Depending on the reduction of the equilibrium moisture content, initial MC and cross section geometry, different sizes of crack depths are expected. Therefore, the horizontal cracks can be qualitatively divided into three groups based on their depth (deep, medium and short), illustrated in Fig. 3. It can be seen that a handful of cracks are significantly deeper than the others and, therefore, associated with the deep group. Cracks, which are not deeper than a few millimeters, are assigned to the short group and all remaining cracks to the medium group. The perpendicular distance from the surface to the crack tip is defined as crack depth $d_{\textrm{c}}$. As investigating the crack depths of the medium group, which is adjusted from outliers, revealed no scientifically significant findings, only the maximum depths are studied in this work. Therefore, to describe the development of the crack pattern, for both the left and the right edge the deepest crack depths ($d_{\textrm{c,l}}^{\textrm{max}}$ and $d_{\textrm{c,r}}^{\textrm{max}}$) are determined. The sum of $d_{\textrm{c,l}}^{\textrm{max}}$ and $d_{\textrm{c,r}}^{\textrm{max}}$ is defined as the maximum total crack depth $d_{\textrm{c}}^{\textrm{max}}$.

#### XFEM

For the determination of the moisture-induced stresses and the crack initiation and crack propagation caused by exceeding the strength limits of wood, XFEM simulations are performed. For the implementation of the multisurface failure criterion, the user element subroutine UDMGINI (Abaqus Documentation 2014) was used.

**Fig. 4:**
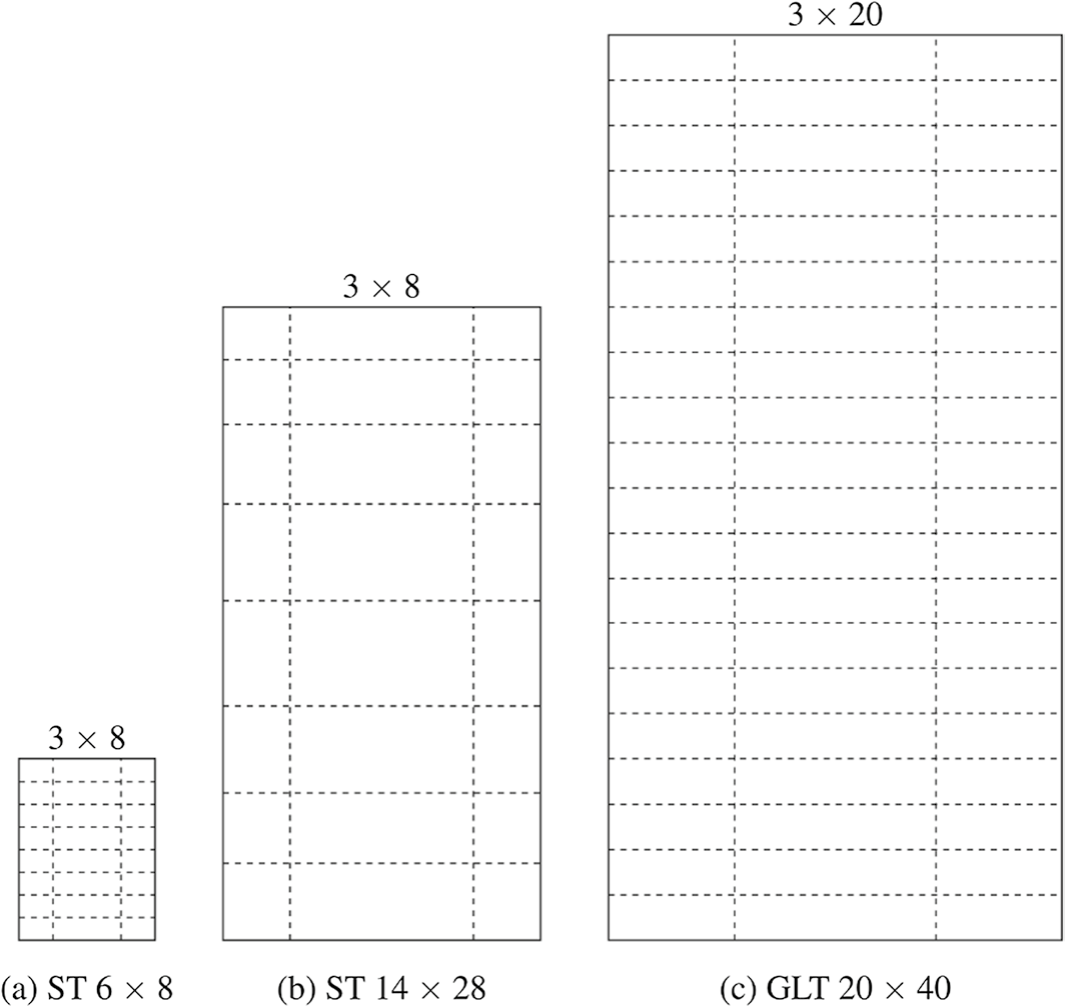
Enrichment region configurations of all cross sections

In XFEM simulations using *Abaqus*, so-called enrichment regions have to be predefined to enable crack initiation and propagation. Only one crack per region is allowed, except in case of crack initiation when multiple elements are fulfilling the limit of the multisurface failure criterion (see "Model for fracture in wood" section) within the same time increment. For the GLT cross section, the enrichment region configuration is based on the study of Autengruber et al. (2021a), where each lamella is defined by two regions, where one is below the other and both are characterized by the same geometry. In case of the ST cross sections, a subdivision of 8 $\times$ 3 (8 rows and 3 columns) is used, which is suitable for severe loads. Figure 4 illustrates the enrichment region configurations used for the simulations of all cross sections.

#### Material properties

For the XFEM simulations, the elastic material tensors of Hofstetter et al. (2005) were utilized, which are based on a clear wood dry density of 420 $\mathrm {kg\,m^{-3}}$ and determined with a continuum micromechanics model. Using these data, moisture-dependent micromechanical parameters were obtained for a range between 3 % and 30 % MC in an 1 % increment interval for spruce (*Picea abies*), as shown in Table 4 in Online Resource 1. For the determination of the material parameters between the MC values, where the tensor components are predefined and the actual values are based on the moisture field, the corresponding values are interpolated. The values for the non-uniform directional moisture and thermal expansion coefficients can be obtained from Table 3 in Online Resource 1.

#### Multisurface failure criterion

The inhomogeneity and orthotropic material properties varying in the direction characteristic for wood also complicate the definition of a strength limit. In Lukacevic et al. (2017), various simulations with different load combinations were used to develop a multisurface failure criterion at the level of annual rings under consideration of multiple Tsai–Wu failure surfaces (Tsai and Wu 1971):

7 $$\begin{aligned}{} & {} f_\textrm{i}^{\textrm{cw}} \left( \mathbf {\sigma } \right) = a_{\textrm{LL,i}} \, \sigma _{\textrm{LL}} + a_{\textrm{RR,i}} \, \sigma _{\textrm{RR}} + a_{\textrm{TT,i}} \, \sigma _{\textrm{TT}} + b_{\textrm{LLLL,i}} \, \sigma ^2_{\textrm{LL}} + b_{\textrm{RRRR,i}} \, \sigma ^2_{\textrm{RR}} \nonumber \\{} & {} \quad + b_{\textrm{TTTT,i}} \, \sigma ^2_{\textrm{TT}} + 2b_{\textrm{RRTT,i}} \, \sigma _{\textrm{RR}} \, \sigma _{\textrm{TT}} + 4b_{\textrm{LRLR,i}} \, \tau ^2_{\textrm{LR}} + 4b_{\textrm{RTRT,i}} \, \tau ^2_{\textrm{RT}} + 4b_{\textrm{TLTL,i}} \, \tau ^2_{\textrm{TL}} \le 1 \end{aligned}$$

**Fig. 5:**
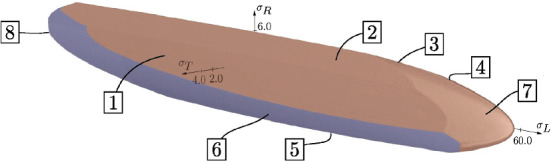
Failure criterion surfaces, presented in the $\sigma _{\textrm{L}}-\sigma _{\textrm{R}}-\sigma _{\textrm{T}}$ stress space from Lukacevic et al. (2017). The surfaces 1,2,3 and 7 define brittle failure

$a_{\textrm{LL,i}}$, $a_{\textrm{RR,i}}, a_{\textrm{TT,i}}$, $b_{\textrm{LLLL,i}}$, $b_{\textrm{RRRR,i}}$, $b_{\textrm{TTTT,i}}$, $b_{\textrm{RRTT,i}}$ , $b_{\textrm{LRLR,i}}$, $b_{\textrm{RTRT,i}}$ and $b_{\textrm{TLTL,i}}$ are the Tsai–Wu parameters as shown in Table 5 in Online Resource 1. This enables the description of brittle (cracking) and ductile (plastic) failure. If brittle failure occurs, the orientation of initiated cracks is determined based on the corresponding failure surfaces’ normal vector. The eight failure surfaces define the direction-dependent strength, shown in Fig. 5, where the surfaces 1,2,3 and 7 consider brittle failure. As a reference, the longitudinal tensile strength is about 56 MPa, the radial one is approximately 5 MPa, and the tangential one is about 2 MPa. During the following evaluation of the results, the failure surfaces do not vary in terms of moisture reduction, although the strength depends on the moisture level, as also assumed in Autengruber et al. (2021a). During the simulations, crack initiation occurs along the cross sections’ edges where a lower MC level is given at the time of cracking, and therefore, a higher strength can be assumed, making the resulting error of a constant strength negligibly small.

### Geometries

**Fig. 6:**
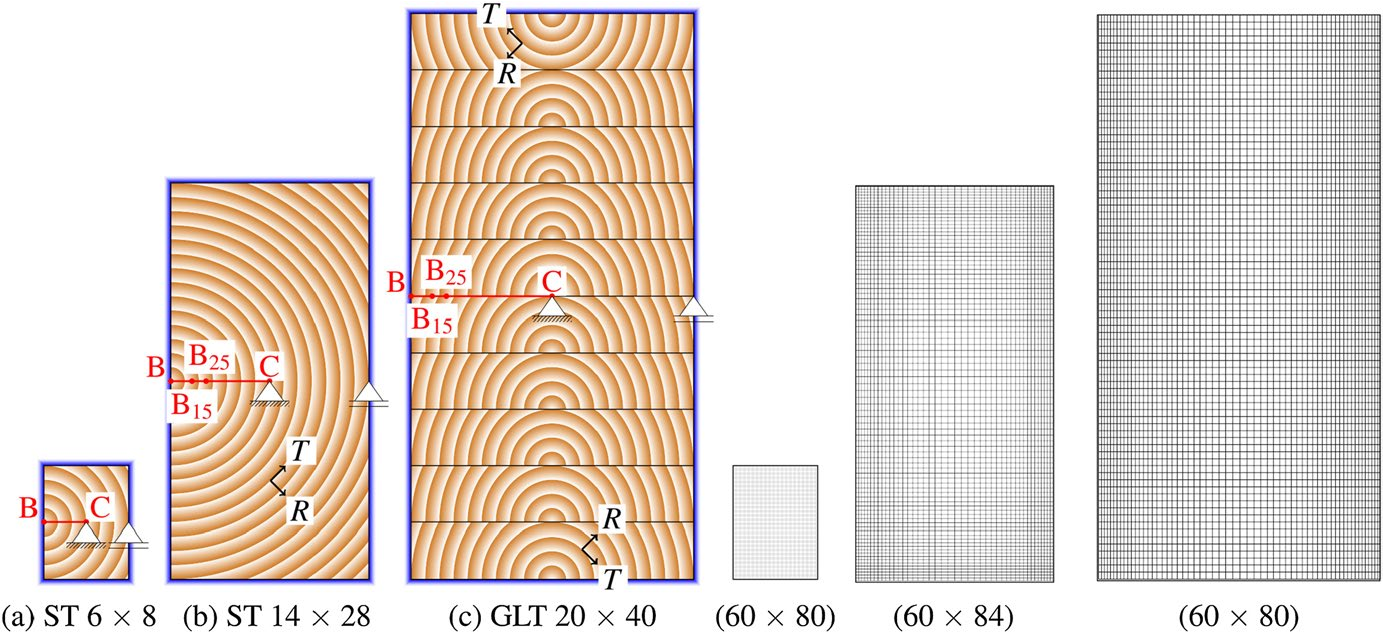
Illustration of the geometric boundary conditions, the locations of piths as well as the definition of local coordinate systems and of finite element node reference points: B, $\textrm{B}_{15}$, $\textrm{B}_{25}$ and C, where $\textrm{B}_{15}$ and $\textrm{B}_{25}$ are 15 mm and 25 mm, respectively, distant to the boundary. $\textrm{B}_{15}$ and $\textrm{B}_{25}$ are required for the moisture gradient definition in Sect. "Relation of crack depth and moisture gradient". The frame along the cross section edges illustrates where the boundary condition is applied during the moisture field simulation. In addition, the mesh configurations used for the simulations of all cross sections are shown.

The simulations are performed on three different rectangular, commonly used cross sections to investigate the influence of the size on the resulting crack development. Two of them are solid timber (ST), and one is a glued laminated timber (GLT). The used cross sections are ST 6 $\times$ 8, ST 14 $\times$ 28 and GLT 20 $\times$ 40 (width $\times$ height), where dimensions are given in centimeters. While the pith of the ST cross sections is located in the middle of the left edge, the piths in the GLT cross section are positioned in the middle of the bottom side of the lamellas, as shown in Fig. 6. The lamella on the top is flipped, and the height of the lamellas is 4 cm. It is assumed that the glue lines between the lamellas do not influence the moisture transport, since the diffusion characteristics of the assumed adhesive melamine hardly differ from solid timber (see Tab. 8 of Volkmer et al. , 2012). The orientation of the lamellas was chosen under consideration of common production standards (ÖNORM EN 14080 2013), and the locations of the piths were selected as in Autengruber et al. (2021a) to expect the highest cracking potential. In Autengruber et al. (2021a), the crack patterns of ST and GLT cross sections were analyzed depending on the pith location.

In longitudinal direction, the model dimension was chosen with one element (1 mm) to model plain-strain conditions. An overview of the remaining dimensions of the mesh can be seen in Table 1, and the mesh configurations used for the simulations are displayed in Fig. 6. The geometric boundary conditions are defined by a hinged support in the center point and a roller support in the middle of the right edge of the cross section (see Fig. 6). As plane-strain conditions are assumed, no deformations out-of-plane occur. Furthermore, it is assumed that, initially, no eigenstresses from, e.g., the production process, are present.

**Table 1:** Mesh configurations of the cross sections

	ST 6 $\times$ 8	ST 14 $\times$ 28	GLT 20 $\times$ 40
Number of elements	60 $\times$ 80	60 $\times$ 84	60 $\times$ 80
Width of elements (mm)	1	2-4	2-5
Height of elements (mm)	1	2-5	5

## Results

In this section, the process of crack initiation and propagation caused by the single-step reductions of the RH is exemplarily described, followed by mesh studies. Afterward, the developments of crack depths in case of different single-step reductions in RH over time are displayed and compared considering geometry and initial MC. Furthermore, the relation between crack depth and MC gradients is analyzed and subsequently examined in terms of geometry and initial MC. In addition, the effect of linear RH reductions over time is studied.

**Fig. 7:**
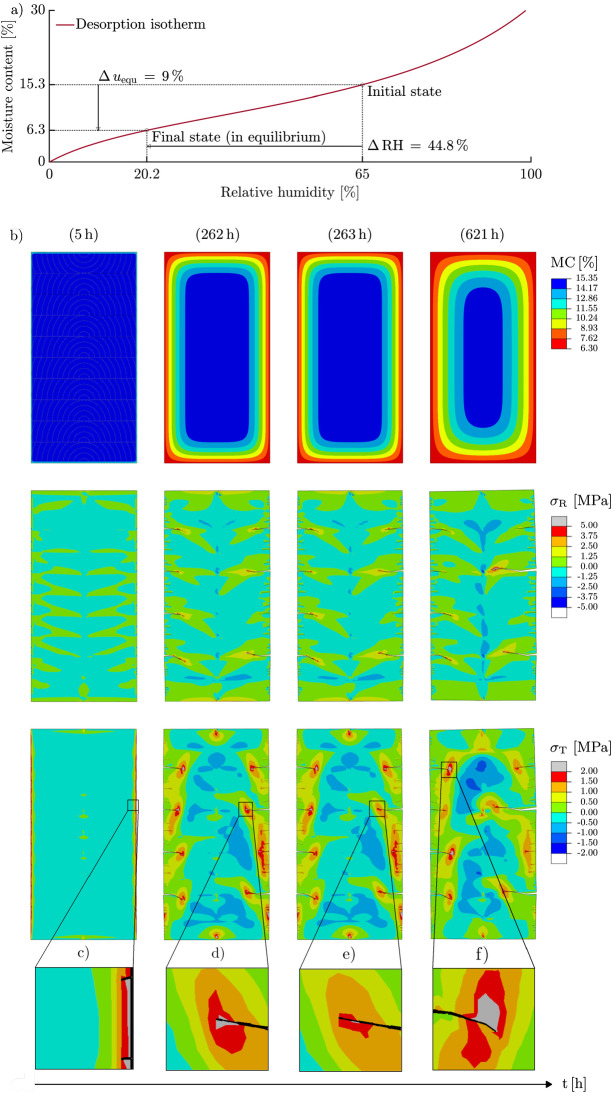
Schematic description of the cracking process. (a) Reducing the relative humidity considering the desorption isotherm (Frandsen et al. 2007b) at the boundary result in (b) moisture content field adaptions. The moisture-induced stresses, such as $\sigma _{\textrm{R}}$ and $\sigma _{\textrm{T}}$, lead to (c) initiation or (d–e) propagation of cracks, if the multisurface failure criterion (see "Model for fracture in wood" section) is violated. (f) Cracking is completed when the resulting stresses no longer increase in such a way that the criterion is violated. (For better perceptibility, the reader is referred to the web version of this article)

To improve the understanding of the crack simulation process, all its steps are first shown in Fig. 7, where the RH is reduced from 65 % (initial state) to 20.2 % (final state), resulting in a $\Delta u_{\textrm{equ}}$ of 9 % for the GLT 20 $\times$ 40 (see Fig. 7a). The shown drying scenario corresponds to the worst case in indoor climate conditions (20 % RH) described in "Introduction" section. As soon as the moisture simulation is initiated, the initial conditions adapt to the single-step reduction of the RH leading to a moisture field (see Fig. 7b). In the subsequent analysis, the XFEM simulation, moisture-induced stresses caused by the loading of the previously calculated moisture distribution are determined per increment. During the stress analysis, in all integration points, the multisurface failure criterion is evaluated, as presented in "Model for fracture in wood" section. If the criterion is fulfilled, a crack initiates (see Fig. 7c, 5h) or propagates (see Fig. 7d and e, 262 h and 263 h). In Fig. 7, only $\sigma _{\textrm{R}}$ and $\sigma _{\textrm{T}}$ are shown, as they mainly contribute to the violation of the failure criterion. The crack formation is completed when the resulting stresses in the remaining elements no longer increase in such a way that the criterion is violated (see Fig. 7 f, 621 h).

### Mesh study

**Fig. 8:**
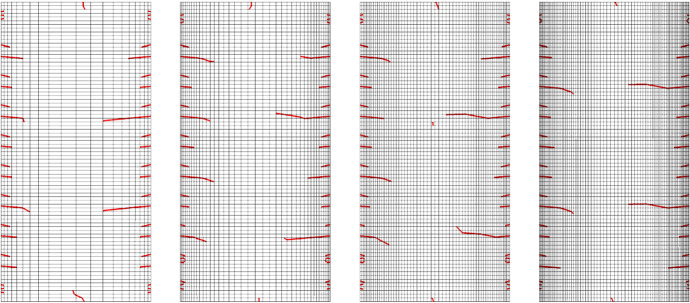
Different mesh configurations influence the crack pattern. Width: 20/40/60/80 elements; height: 80 elements. Selected mesh: 60 elements

**Fig. 9:**
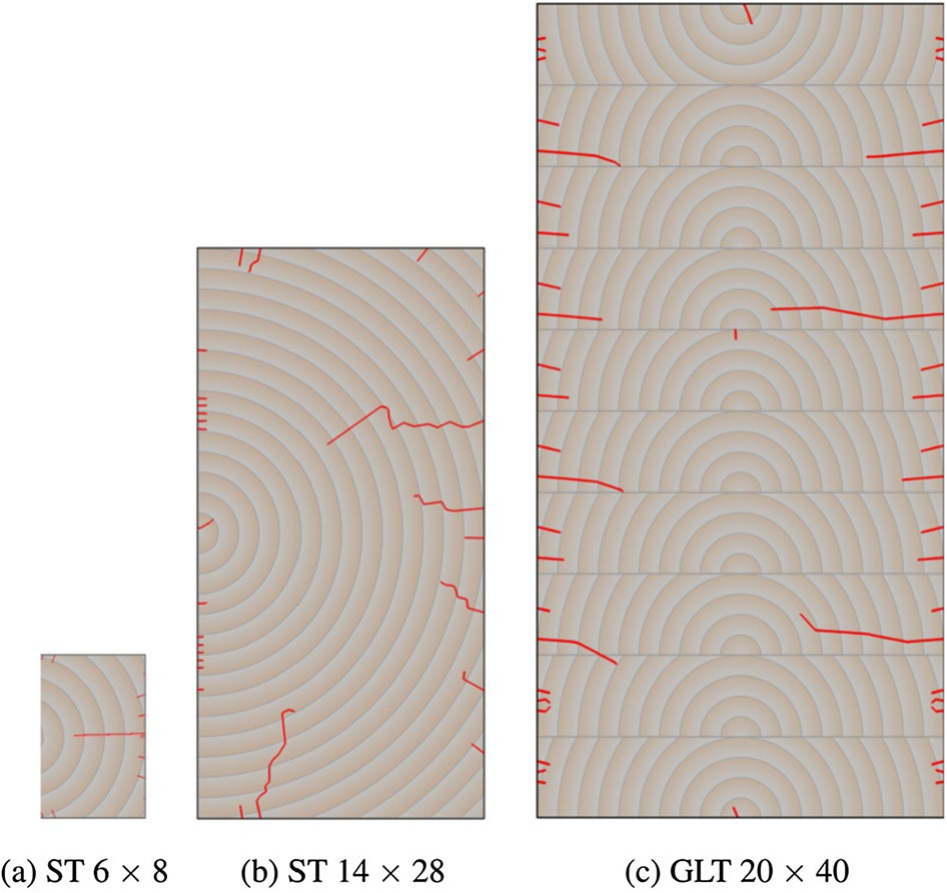
Overview of the cross section dependent crack patterns at the end of the simulation (t = 30 d), when the initial MC is reduced from 15.3 % to 2.3 %. In addition, the pith location and the annual rings of the cross sections are illustrated

In order to evaluate the effect of the mesh size on the simulation results, different mesh configurations were investigated, as shown in Fig. 8. They were analyzed for all cross sections, but the procedure is exemplified for the cross section GLT 20 $\times$ 40. As cracks predominantly develop along the width, the element size was left constant with 80 elements in the height direction. Additionally, the width of the elements decreases closer to the edges to determine the crack development in the area of initiation more precisely. Comparing the different mesh configurations reveals a similar basic crack pattern, since the locations of the initiated cracks are close to each other and average depths are almost equivalent. However, the depth of the deepest cracks varies, making this feature suitable to determine the final mesh, which was identified by analyzing the deepest cracks at predefined points in time (see "Cracking behavior of different cross sections" section). With increasing number of elements, the deepest crack depth should not vary significantly, as the moisture-induced stress fields deviate less with mesh refinement. Since the deepest crack depths of the mesh consisting of 60 elements along the width differ only slightly compared to 80 elements in the width, the former configuration was chosen for all further simulations. As deep vertical cracks also occur for the ST 14 $\times$ 28 (see Fig. 9), the height of the elements was varied, decreasing closer to the top or bottom.

### Cracking behavior of different cross sections

After examining the mesh size and enrichment region configurations, the cross section depending crack behavior at the end of the simulations is compared to highlight differences regarding different geometries. In the following example an MC reduction from 15.3 % to 2.3 % ($\Delta u_{\textrm{equ}} =$ 13 %) is shown for all cross sections, as this led to characteristic crack formations for all of them. The crack patterns differ depending on the pith location and geometry. For the ST cross sections, cracks develop on the top, bottom, and right edge of the cross section, while the left edge remains predominantly intact due to the location of the pith, which is situated at the middle of the left edge. Nevertheless, also differences are noticeable, such as the location of the deepest cracks. While for the ST 6 $\times$ 8 the deepest crack is located at the middle height of the cross section, for the ST 14 $\times$ 28 the deepest one can be observed at three-quarters of the height. In case of lower drying loads ($\Delta u_{\textrm{equ}} \le$ 11 %) for the ST 14 $\times$ 28, the deepest crack is also at the middle height of the cross section. Besides, deep vertical cracks on both the top and bottom of the ST 14 $\times$ 28 occur, which is related to the height-to-width ratio as additional simulations confirmed. Further investigations are required to determine all aspects of the deep vertical crack propagation. In contrast to the ST, the GLT cross section shows a different cracking behavior. For the GLT cross section, where the piths are positioned in the middle of the bottom side of the lamellas, cracks emerge in each lamella at both edges. Most lamellas have one deeper crack and two shorter cracks, but also lamellas with fewer cracks occur.

#### $d_{\textrm{c}}^{\textrm{max}}$ development

After the comparison of different crack formations depending on the cross section at the end of the simulations, an overview of the crack development for all cross sections and single-step RH configurations shown in Fig. 2 is given by plotting $d_{\textrm{c}}^{\textrm{max}}$ over time for varying $\Delta u_{\textrm{equ}}$ and different initial MCs. $d_{\textrm{c,l}}^{\textrm{max}}$ and $d_{\textrm{c,r}}^{\textrm{max}}$ were documented at predefined points in time (0.25 d, 0.5 d, 1 d, 2 d, 3 d, 5 d, 10 d, 15 d, 20 d and 30 d), where $d_{\textrm{c}}^{\textrm{max}}$ was subsequently determined. Frech (1998) pointed out that $d_{\textrm{c,l}}^{\textrm{max}}$ and $d_{\textrm{c,r}}^{\textrm{max}}$ of 15 mm to 30 mm are still acceptable for the load-bearing capacity of GLT girders, but attention should be paid to the shape of the timber member and to the type of load when designing timber structures. Therefore, a crack depth of 2 $\times$ 15 mm is highlighted in the figures of the GLT cross section. As cracks also compromise the load-bearing capacity of ST cross sections, a depth of 15 mm is marked due to the one-sided crack development under the given conditions.

**Fig. 10:**
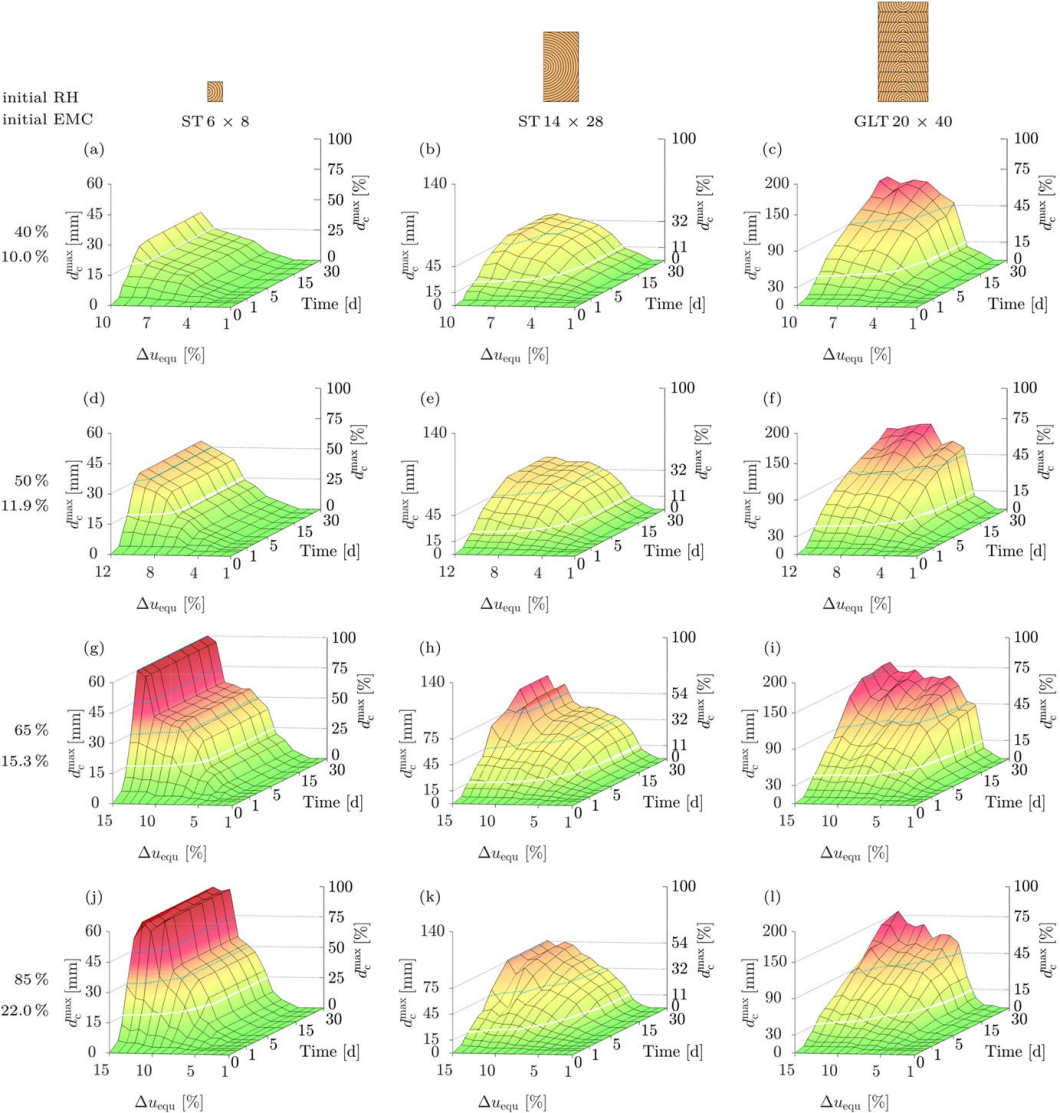
Development of the maximum total crack depth $d_{\textrm{c}}^{\textrm{max}}$ (absolute and in percent of the width) for all cross sections with initial moisture contents of 10.0 % (a,b,c), 11.9 % (d,e,f), 15.3 % (g,h,i) and 22.0 % (j,k,l) under various reductions of the equilibrium moisture content $\Delta u_{\textrm{equ}}$ (from 1 % to 10 %, 12 % or 15 %) over 30 days. The white lines show the limit (ST: 15 mm, GLT: 30 mm), above which attention should be paid to the shape of the timber member and to the type of load when designing timber structures according to Frech (1998). The dotted lines improve perceptibility of the $d_{\textrm{c}}^{\textrm{max}}$ values

Figure 10 shows the development of $d_{\textrm{c}}^{\textrm{max}}$ under different conditions, illustrated in Fig. 2, where both are given in mm as well as in percent (cracked width). First, all effects caused by a change in drying load $\Delta u_{\textrm{equ}}$ are specified, exemplarily with an initial MC of 15.3 % for all cross sections (see Fig. 10(g) to (i)) and displayed in Table 2. Increasing $\Delta u_{\textrm{equ}}$ causes an earlier crack initiation as well as a faster development of $d_{\textrm{c}}^{\textrm{max}}$ caused by quicker stress development. Furthermore, the crack development ends at different points in time, where with increasing $\Delta u_{\textrm{equ}}$ crack propagation comes to a halt later. (In few cases, the theoretical crack formation can even last longer than 30 d.)

**Table 2:** Overview of all effects caused by a change in drying load $\Delta u_{\textrm{equ}}$: Difference in the maximum total crack depth $d_{\textrm{c}}^{\textrm{max}}$ at a certain point in time (exemplarily after 1 d) and initial as well as final point in time of crack development. “−” means that no crack initiates

Cross section	Crack initiating (d)			$d_{\textrm{c}}^{\textrm{max}}$ (mm)			Cracking finished (d)		
	3 %	4 %	7 %	7 %	8 %	9 %	3 %	4 %	5 %
ST 6 $\times$ 8	−	1	0.25	8	10	12	−	1	2
ST 14 $\times$ 28	1	0.50	0.25	9	11	12	5	10	15
GLT 20 $\times$ 40	2	0.50	0.25	12	17	22	5	10	30

In the following, the reason for different points time when cracking ends is analyzed more closely. The smaller the width of the cross section, the earlier the reduction of the MC in the center occurs. For all cross sections, the MC decreases from both sides toward the center at the same rate until the center is reached. From this point on, the MC reduction in the center is accelerated due to the influence of the opposite edge, as the reduction of the RH affects all edges. If the boundary condition is only applied to one edge, the MC reduction in the center would not be accelerated, as described in Svensson et al. (2011). Hence, crack development of the ST 6 $\times$ 8 stops earlier due to reduced moisture differences preventing cracking.

In addition, it is particularly striking that the minimum $\Delta u_{\textrm{equ}}$ to initiate cracking differs for the cross sections (see Fig. 10(g) to (i)). While for the ST 14 $\times$ 28 and the GLT 20 $\times$ 40, a $\Delta u_{\textrm{equ}}$ of 3 % leads to crack formation, for the ST 6 $\times$ 8, a $\Delta u_{\textrm{equ}}$ of 4 % is necessary. Only in case of an initial MC of 22.0 % a $\Delta u_{\textrm{equ}}$ of 4 % is required for crack initiation to occur for the ST 14 $\times$ 28. It follows that increasing the size of the cross section leads to comparatively earlier crack initiation. Since the moisture flux through the surface of all cross sections is equal during the first hours, the size of the larger cross sections leads to larger moisture-induced stresses as the MC in the center is only hardly reduced, causing greater differences in MC.

Besides, it can be observed for all cross sections that $d_{\textrm{c}}^{\textrm{max}}$ after 30 d does not always increase with increasing $\Delta u_{\textrm{equ}}$, but seems to converge toward a limit, which is further investigated in Sect. "Relation of crack depth and moisture gradient".

Finally, the effect of initial MC on $d_{\textrm{c}}^{\textrm{max}}$ development is studied, with all particularly striking effects for all cross sections and initial MC levels shown in Table 3. Increasing the initial MC results in a tendency of later crack initiation and a tendency for longer-lasting crack propagation. Both can be explained by the nonlinear shape of the desorption isotherm (see Fig. 7(a)) as well as the nonlinear behavior of the sorption rate.

**Table 3:** Overview of all effects caused by changing the initial MC: Difference in initial as well as final point in time of crack development

Cross section	Crack initiating between ... (d)				Cracking finished between ... (d)			
	10.0 %	11.9 %	15.3 %	22.0 %	10.0 %	11.9 %	15.3 %	22.0 %
ST 6 $\times$ 8	0.25-0.50	0.25-0.50	0.25-1	0.25-2	0.50-3	0.50-3	1-3	2-10
ST 14 $\times$ 28	0.25-0.50	0.25-1	0.25-1	0.25-2	10-20	10-20	10-20	10-25
GLT 20 $\times$ 40	0.25-1	0.25-1	0.25-2	0.25-5	5-30	10-30	10-30	10-30

### Relation of crack depth and moisture gradient

In "Cracking behavior of different cross sections" section, the crack depth depending on time and $\Delta u_{\textrm{equ}}$ is displayed and analyzed. Now, we want to find a relation between the crack depth and the moisture distribution to predict crack depths. Dietsch et al. (2015) showed that a considerable amount of cracks in timber constructions are related to moisture changes, which can be described with moisture gradients. Those were evaluated between 15 mm and 25 mm from the surface (Svensson et al. 2011). In this paper, a similar approach is used, and therefore, the moisture gradient is defined as the difference in MC between two points P$_1$ and P$_2$ divided by their distance $(\Delta u / \Delta x)_{\mathrm {P_1P_2}}$. Autengruber et al. (2021a) indicated that the deepest cracks are related to the greatest difference in MC between the center and the boundary of the cross section. As Dietsch et al. (2015) and Autengruber et al. (2021a) show that different positions used to determine moisture differences and gradients are suitable for the description of crack behavior, their influence is also analyzed. They are positioned between the boundary (B) and the center (C) (see Fig. 6), where the marked positions B$_{\textrm{15}}$ and B$_{\textrm{25}}$ are 15 mm and 25 mm distant to the surface, respectively. As B$_{15}$ and B$_{25}$ are not located on a finite element node compared to B and C, the nodes closest to them define the points for the moisture gradient (see Table 4). Three configurations of $(\Delta u / \Delta x)_{\mathrm {P_1P_2}}$ are investigated: the moisture gradient between B and C $(\Delta u / \Delta x)_{\textrm{BC}}$, between B$_{15}$ and B$_{25}$ $(\Delta u / \Delta x)_{\mathrm {B_{15}B_{25}}}$ and between B$_{15}^{\textrm{B}}$ and B$_{15}^{\textrm{C}}$ $(\Delta u / \Delta x)_{\mathrm {B_{15}^{B}B_{15}^{C}}}$. In case of $(\Delta u / \Delta x)_{\mathrm {B_{15}^{B}B_{15}^{C}}}$, the two closest finite element nodes to B$_{\textrm{15}}$, where one is nearer to B (B$_{15}^{\textrm{B}}$) and the other to C (B$_{15}^{\textrm{C}}$) are used for the moisture gradient’s definition (see Table 4). As the diffusion coefficients in radial and tangential direction are assumed to be equal, the values of the horizontal nodes, which have the same horizontal distance to C, are identical to the left and right of C, respectively, so the position of B does not depend on whether it is located on the left or right edge.

**Table 4:** Horizontal distance from B (boundary) (see Fig. 6) for the positions B$_{15}$, B$_{25}$, B$_{15}^{\textrm{B}}$ and B$_{15}^{\textrm{C}}$, which relate to locations of finite element nodes, used for the definition of the moisture gradient for all cross sections

Cross section	Horizontal distance (mm)			
	B$_{15}$	B$_{25}$	B$_{15}^{\textrm{B}}$	B$_{15}^{\textrm{C}}$
ST 6 $\times$ 8	15.00	25.00	14.00	15.00
ST 14 $\times$ 28	14.88	25.05	14.88	16.80
GLT 20 $\times$ 40	15.56	26.16	13.12	15.56

#### Approach description

**Fig. 11:**
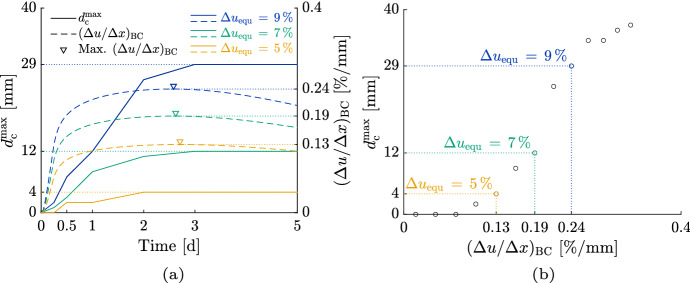
Relation between the moisture gradient between the points B (boundary) and C (center) $(\Delta u / \Delta x)_{\textrm{BC}}$ (see Fig. 6) and the maximum total crack depth ($d_{\textrm{c}}^{\textrm{max}}$) for the ST 6 $\times$ 8 in case of an exemplary initial MC of 15.3 %. In (a) the developments of $d_{\textrm{c}}^{\textrm{max}}$ and $(\Delta u / \Delta x)_{\textrm{BC}}$ in case of three reductions of the equilibrium moisture content $\Delta u_{\textrm{equ}}$ (5 %, 7 % and 9 %) are shown. In (b), the maxima of $d_{\textrm{c}}^{\textrm{max}}$ and $(\Delta u / \Delta x)_{\textrm{BC}}$ in case of $\Delta u_{\textrm{equ}}$ reductions from 1 % to 13 % are displayed, illustrating the relation between $d_{\textrm{c}}^{\textrm{max}}$ and $(\Delta u / \Delta x)_{\textrm{BC}}$

Before the investigation of the dependency of the point’s locations, an approach to describe the relation between $d_{\textrm{c}}^{\textrm{max}}$ and, exemplary, $(\Delta u / \Delta x)_{\textrm{BC}}$ is given, where the result is shown in Fig. 11. Both the developments of $d_{\textrm{c}}^{\textrm{max}}$ and $(\Delta u / \Delta x)_{\textrm{BC}}$ are given in case of three different $\Delta u_{\textrm{equ}}$ (5 %, 7 % and 9 %) with an exemplary initial MC of 15.3 % for the ST 6 $\times$ 8 (see Fig. 11(a)). It can be seen that for each point in time a $(\Delta u / \Delta x)_{\textrm{BC}}$ can be connected to a $d_{\textrm{c}}^{\textrm{max}}$. Since the maximum of $d_{\textrm{c}}^{\textrm{max}}$ that occurs after an installation is essential for the design of timber structures, we correlate the maxima of $d_{\textrm{c}}^{\textrm{max}}$ and $(\Delta u / \Delta x)_{\textrm{BC}}$.

Observing the development of $(\Delta u / \Delta x)_{\textrm{BC}}$ and $d_{\textrm{c}}^{\textrm{max}}$ shows that the crack depth can increase after $(\Delta u / \Delta x)_{\textrm{BC}}$ reached its maximum. While in case of a $\Delta u_{\textrm{equ}}$ of 7 % and 9 % the maximum of $(\Delta u / \Delta x)_{\textrm{BC}}$ is observed before the end of crack propagation, in case of a $\Delta u_{\textrm{equ}}$ of 5 % the increase of $(\Delta u / \Delta x)_{\textrm{BC}}$ comes to a halt after the crack propagation stops. The differences in case of 7 % and 9 % are related to a still ongoing moisture flux in the cross section inducing sufficient internal stress to cause crack propagation. Besides, with increasing $\Delta u_{\textrm{equ}}$ the maximum of $(\Delta u / \Delta x)_{\textrm{BC}}$ is observed earlier. While for a $\Delta u_{\textrm{equ}}$ of 9 % the maximum is reached after 62 h, for a $\Delta u_{\textrm{equ}}$ of 5 % the increase of $(\Delta u / \Delta x)_{\textrm{BC}}$ ends after 65 h. The time gap of the maxima is related to an earlier reduction of the MC in the cross section’s center.

In Fig. 11(b), the maxima of $(\Delta u / \Delta x)_{\textrm{BC}}$ and $d_{\textrm{c}}^{\textrm{max}}$ in case of $\Delta u_{\textrm{equ}}$ reductions from 1 % to 13 % are correlated, where $\Delta u_{\textrm{equ}}$ = 14 % and $\Delta u_{\textrm{equ}}$ = 15 % are excluded, as they are assessed as outliers. The resulting data show a pattern characterized by exponential growth until a certain point. The so-called Gompertz function can describe the growth behavior, characterized by exponential growth converging to a limit with few parameters. The Gompertz function is defined as

8 $$\begin{aligned} \begin{aligned} d_{\textrm{c}}^{\textrm{max}} \left( (\Delta u / \Delta x)_{\mathrm {P_1P_2}} \right) = g^{\textrm{max}} e^{-g^{\textrm{l}} e^{-g^{\textrm{g}} \, (\Delta u / \Delta x)_{\mathrm {P_1P_2}}}} \end{aligned} \end{aligned}$$

with $d_{\textrm{c}}^{\textrm{max}}$ as the maximum total crack depth in (mm), the moisture gradient between two points P$_1$ and P$_2$ $(\Delta u / \Delta x)_{\mathrm {P_1P_2}}$ in (%/mm) as well as $g^{\textrm{max}}$, $g^{\textrm{l}}$ and $g^{\textrm{g}}$ as the function parameters, which depend on the cross section, initial MC and point in time and are characterized by different properties. $g^{\textrm{max}}$ describes the asymptote, and therefore, the maximum expected $d_{\textrm{c}}^{\textrm{max}}$, $g^{\textrm{l}}$ sets the displacement of the location along the horizontal axis and $g^{\textrm{g}}$ is the growth rate at the inflection point.

#### Effect of Moisture gradient configuration on relation

**Fig. 12:**
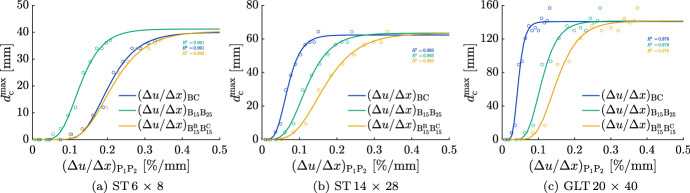
Development of the maximum total crack depth $d_{\textrm{c}}^{\textrm{max}}$ depending on the moisture gradient between two points P$_1$ and P$_2$ $(\Delta u / \Delta x)_{\mathrm {P_1P_2}}$ comparing the influence of the positions of P$_1$ and P$_2$ (B$_{\textrm{15}}$, B$_{\textrm{25}}$, B$_{15}^{B}$ and B$_{15}^{C}$) with an initial moisture content of 15.3 % for all cross sections. The positions are located between the boundary (B) at the middle of the edge and the center (C) (see Fig. 6), where their exact positions can be obtained from Table 4. The data marked with circles represent the maxima of $d_{\textrm{c}}^{\textrm{max}}$ correlated to the maxima of the corresponding $(\Delta u / \Delta x)_{\mathrm {P_1P_2}}$ per equilibrium MC reduction $\Delta u_{\textrm{equ}}$ (see Fig. 11)

Figure 12 shows the relation between $d_{\textrm{c}}^{\textrm{max}}$ and the three configurations $(\Delta u / \Delta x)_{\textrm{BC}}$, $(\Delta u / \Delta x)_{\mathrm {B_{15}B_{25}}}$ and $(\Delta u / \Delta x)_{\mathrm {B_{15}^{B}B_{15}^{C}}}$ for an initial MC of 15.3 % for all cross sections. For each configuration of $(\Delta u / \Delta x)_{\mathrm {P_1P_2}}$, the best-fitting Gompertz function and corresponding coefficient of determination were calculated and displayed. It can be observed that the coefficients of determination are all above 0.97 and the curves fit the data well. Therefore, moisture gradients can be related to moisture-induced stresses and, consequently, to $d_{\textrm{c}}^{\textrm{max}}$ depending on the cross section’s size. Due to the small differences of the three configurations, $(\Delta u / \Delta x)_{\textrm{BC}}$ will be used for further investigations on account of a better possible prediction of the MC at the boundary and in the center in case of larger cross sections and regularly changing climate conditions, as described in Autengruber et al. (2021b).

Besides, additional investigations of further $(\Delta u / \Delta x)_{\mathrm {P_1P_2}}$ configurations showed that not all points P$_1$ and P$_2$ between B and C are suitable to describe the crack depth development. For all cross sections, if P$_1$ is at B or between B and B$_{15}$, P$_2$ has to be at least 1.5 cm distant to B. Furthermore, the closer one point is to C, the closer the other should be to B.

#### Influence of initial MC on relation

**Fig. 13:**
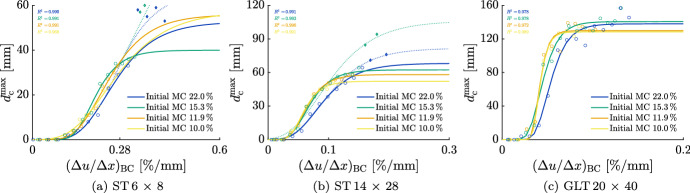
Development of the maximum total crack depth $d_{\textrm{c}}^{\textrm{max}}$ depending on the moisture gradient between the points B (boundary) and C (center) $(\Delta u / \Delta x)_{\textrm{BC}}$ (see Fig. 6) considering different initial moisture contents (MC) for all cross sections. The data marked with circular and diamond symbols represent $d_{\textrm{c}}^{\textrm{max}}$ related to the corresponding moisture gradient per equilibrium MC reduction $\Delta u_{\textrm{equ}}$ (see Fig. 11)

The development of $d_{\textrm{c}}^{\textrm{max}}$ considering moisture differences depends on the initial MC as well. The differences between all cross sections for $(\Delta u / \Delta x)_{\textrm{BC}}$ are displayed in Fig. 13.

In Fig. 13(a) and (b), additional simulation results are shown with diamond symbols. The consideration of these crack depths in the fits would lead to the dotted curves. For the cross section ST 6 $\times$ 8, the additional data show extremal values, as $d_{\textrm{c}}^{\textrm{max}}$ is close to the total width of the cross section. Thus, in these cases, courses would converge to an extrapolated value, which is larger than the total width of the cross section. The continuous lines, on the contrary, show the course resulting from the exclusion of the outliers. Further analyzing the cross section ST 6 $\times$ 8 shows a wide range of possible $d_{\textrm{c}}^{\textrm{max}}$ in case of $(\Delta u / \Delta x)_{\textrm{BC}} \ge 0.28$ (%/mm) with an initial MC of 15.3 %, compared to the cross section ST 14 $\times$ 28.

In case of the cross section ST 14 $\times$ 28, alternative courses of the development of $d_{\textrm{c}}^{\textrm{max}}$ occur as well. While the continuous line shows the course fitting best to the data below a $\Delta u_{\textrm{equ}}$ of 12 % with an initial MC of 15.3 % and 22.0 %, respectively, the dotted curve describes the fitted course of the deepest $d_{\textrm{c}}^{\textrm{max}}$ above a $\Delta u_{\textrm{equ}}$ of 12 %. Apart from the ambiguous course of $d_{\textrm{c}}^{\textrm{max}}$ in case of $(\Delta u / \Delta x)_{\textrm{BC}}$ $\ge 0.1$ (%/mm), the largest $d_{\textrm{c}}^{\textrm{max}}$ occurs with an initial MC of 15.3 %. This can also be determined for the GLT 20 $\times$ 40. Besides, for this cross section, the development of $d_{\textrm{c}}^{\textrm{max}}$ with an initial MC of 10.0 % and 11.9 % is almost identical compared to the other cross sections. For the other initial MCs, the courses of $d_{\textrm{c}}^{\textrm{max}}$  are still similar, like for the ST 14 $\times$ 28, but not identical.

For both ST cross sections no definite development of $d_{\textrm{c}}^{\textrm{max}}$ for $\Delta u_{\textrm{equ}}$ greater than 9 % can be identified, as illustrated by the dotted and the continuous line. Therefore, it is assumed that for ST cross sections branching points in the development of $d_{\textrm{c}}^{\textrm{max}}$ exist. Although for the GLT 20 $\times$ 40 $d_{\textrm{c}}^{\textrm{max}}$ converges to a limit, which is not the width of the cross section, it cannot be ruled out that branching points for GLT cross sections occur as well, since only one cross section was investigated.

In addition, it can be seen that below a $\Delta u_{\textrm{equ}}$ of 11 % $d_{\textrm{c}}^{\textrm{max}}$ is always smaller for an initial MC of 22.0 % compared to the other initial MCs due to slower desorption close to the FSP (Håkansson 1994; Frandsen et al. 2007a).

### Influence of reducing relative humidity linearly over time on crack development

The following analyzes the effect of a linear reduction in RH over time on crack initiation and propagation. As described in "Introduction" section, ventilation systems can influence RH conditions and lead to large $d_{\textrm{c}}^{\textrm{max}}$ values, which should be avoided. However, air conditioning could also be used to ensure a linear reduction in RH over time.

**Fig. 14:**
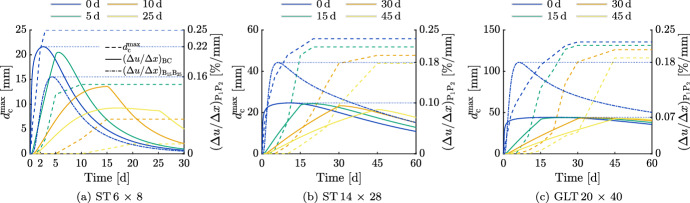
Development of the moisture gradient between the points B and C $(\Delta u / \Delta x)_{\textrm{BC}}$ (see Fig. 6) and the maximum total crack depth ($d_{\textrm{c}}^{\textrm{max}}$) reducing the MC from 15.3 % to 7.3 % over various periods of time from 0 d to 45 d. The legends define different RH reduction time spans. In addition, one development of the moisture gradient between the points $\textrm{B}_{15}$ and $\textrm{B}_{25}$ $(\Delta u / \Delta x)_{\mathrm {B_{15}B_{25}}}$ is given to enable comparison with $(\Delta u / \Delta x)_{\textrm{BC}}$

Therefore, additional simulations were performed to investigate the effect of a continuous reduction of the RH over a longer period of time, rather than an immediate one. The corresponding boundary conditions were decreased from 65.0 % RH to 25.2 % RH over different time spans ranging between 0 d and 45 d, depending on the cross section, which causes a theoretical reduction of the MC from 15.3 % to 7.3 %. The results are displayed in Fig. 14, where the continuous line shows the development of $(\Delta u / \Delta x)_{\textrm{BC}}$, the dash-dotted line represents $(\Delta u / \Delta x)_{\mathrm {B_{15}B_{25}}}$ and the dashed curve illustrates the increase of $d_{\textrm{c}}^{\textrm{max}}$. The maxima of $d_{\textrm{c}}^{\textrm{max}}$, $(\Delta u / \Delta x)_{\textrm{BC}}$ and $(\Delta u / \Delta x)_{\mathrm {B_{15}B_{25}}}$ are summarized in Table 6 in Online Resource 1. Differences in the development of $d_{\textrm{c}}^{\textrm{max}}$ can be observed between the cross sections. While for the ST 6 $\times$ 8, $d_{\textrm{c}}^{\textrm{max}}$ decreases significantly the longer the reduction of the RH lasts, for the ST 14 $\times$ 28 and the GLT 20 $\times$ 40, $d_{\textrm{c}}^{\textrm{max}}$ is hardly reduced, even though the decrease of the RH lasts up to 45 d. It follows that the portion of $d_{\textrm{c}}^{\textrm{max}}$, which can be reduced by a linear reduction in RH over time, decreases with increasing cross section size. This is caused by the earlier reduction of the MC in the center of smaller cross sections, as described in "Cracking behavior of different cross sections" section.

Therefore, damage can be prevented by reducing the RH over a longer period of time through the appropriate use of ventilation systems. However, it appears that only for small cross sections the approach of a continuous reduction of the RH is reasonable due to the quicker adaption of the cross sections’ moisture field. Since a slower reduction of the RH seems to hardly influence the cracking behavior of larger cross sections, it is suggested to reduce the initial MC during production to avoid crack initiation and propagation. After a certain amount of time, a continuous reduction of the RH would slowly reduce the MC without causing deep $d_{\textrm{c}}^{\textrm{max}}$, but due to the long duration, it would not be suitable for practical application. As an example for the GLT 20 $\times$ 40, a continuous reduction of the RH over 300 d would result in a maximum $d_{\textrm{c}}^{\textrm{max}}$ of 36 mm.

In addition to the differences in $d_{\textrm{c}}^{\textrm{max}}$ development, the occurrence of the maximum of $(\Delta u / \Delta x)_{\textrm{BC}}$, where the RH is reduced over 25 d, differs in case of the ST 6 $\times$ 8 (see Fig. 14(a)). The maxima of all courses can be observed as soon as the reduction of the RH is completed, but when reducing the RH over 25 d, its maximum occurs after about 15 d, although the RH still changes. This can also be explained by the earlier decrease of the MC in the center of the cross section, but in this case, the reduction rate of the MC in C becomes larger than the one at B. Since it can only be observed for the ST 6 $\times$ 8, it is assumed that a small width is required for this to occur. It can already be noticed when the RH is reduced over 17.5 d, as additional simulations confirmed.

The development of $(\Delta u / \Delta x)_{\textrm{BC}}$ for all cross sections shows increasing maxima with decreasing size of the cross section. However, analyzing $(\Delta u / \Delta x)_{\mathrm {B_{15}B_{25}}}$ reveals that its maximum increases with greater dimensions of the cross section, as the MC in C is reduced earlier in smaller cross sections. This was also discovered in Chen et al. (2019) where timber with circular cross sections was dried in a climate chamber. Therefore, it can be stated that with greater size of the cross section the occurring moisture difference increases. Due to the influence of the cross section’s width, this behavior is reversed in case of $(\Delta u / \Delta x)_{\textrm{BC}}$.

Finally, the time interval between the maxima of $(\Delta u / \Delta x)_{\textrm{BC}}$ and of $d_{\textrm{c}}^{\textrm{max}}$ varies depending on the cross section, as can be seen in Fig. 14. Increasing the size of the cross sections leads to a longer time interval between the extremes. This is related to the longer-lasting moisture flux of larger cross sections. In case of the ST 6 $\times$ 8, the moisture flux rapidly decreases after the maximum of $(\Delta u / \Delta x)_{\textrm{BC}}$ due to the influence of the opposite edge (Svensson et al. 2011) (see "Cracking behavior of different cross sections" section), and, thus, the maximum of $d_{\textrm{c}}^{\textrm{max}}$ occurs shortly thereafter. For the larger cross sections, the moisture field continues to adapt after the maximum of $(\Delta u / \Delta x)_{\textrm{BC}}$, which is why the development of $d_{\textrm{c}}^{\textrm{max}}$ comes to a halt not until about 45 d.

## Discussion

The results show that moisture reductions influence crack initiation and propagation depending on the geometry and initial MC. As shown in this paper, the determination of $d_{\textrm{c}}^{\textrm{max}}$ is possible for wooden cross sections exposed to indoor climate conditions, but the approach is not easily applicable by timber engineers. Therefore, the determination of $d_{\textrm{c}}^{\textrm{max}}$ is simplified in a further step.

### Simplified crack depth determination for indoor climate conditions

**Fig. 15:**
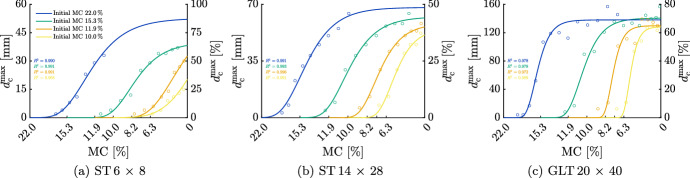
Induced maximum total crack depth $d_{\textrm{c}}^{\textrm{max}}$, absolute and in percent of the width, caused by reduction of the moisture content (MC). The data marked with circular symbols represent $d_{\textrm{c}}^{\textrm{max}}$ related to the corresponding MC per equilibrium MC reduction $\Delta u_{\textrm{equ}}$

The results show that drying can lead to deep cracks if large moisture loads occur. As mentioned in Sect. "Introduction", the RH in case of indoor conditions can be better quantified compared to outdoor climates (Bertolin et al. 2015; Ferdyn-Grygierek 2014; Nguyen et al. 2014). To evaluate the possible $d_{\textrm{c}}^{\textrm{max}}$ in buildings based on the simulation results, it is assumed that the timber components used for wooden structures are protected from weather conditions during transport to the construction site until the start of operation. This results in similar initial conditions in the cross section. To determine which decrease relative to the initial MC leads to which $d_{\textrm{c}}^{\textrm{max}}$, the crack depth development is shown in Fig. 15. The displayed curves are identical to the continuous curves of Fig. 13. Although the desorption isotherm is idealized below an MC of 5 %, the corresponding data and segments of the curve are added to comprehend the development above 5 % MC. To improve the comparability, the resulting potential $d_{\textrm{c}}^{\textrm{max}}$ is displayed in Table 5 given in relation to the total width of the cross section. As can be seen in both Fig. 15 and Table 5, the initial MC significantly influences $d_{\textrm{c}}^{\textrm{max}}$. While with an initial MC of 10.0 %, $d_{\textrm{c}}^{\textrm{max}}$ is relatively low with a maximum of about 12 % of the cross-sectional width, with an initial MC of 11.9 %, the depth of the cracks increases. For the ST 6 $\times$ 8, $d_{\textrm{c}}^{\textrm{max}}$ is almost 7 %, but for the ST 14 $\times$ 28 about 26 % of the width is cracked. The largest $d_{\textrm{c}}^{\textrm{max}}$, which adds up to approximately 54 %, can be observed in case of the GLT 20 $\times$ 40. The significant influence of the initial MC can be seen at a level of 15.3 %: While for the ST 6 $\times$ 8, a crack propagation between 28 % and 48 % of the width is possible, for the ST 14 $\times$ 28, $d_{\textrm{c}}^{\textrm{max}}$ increases to roughly 40 %. However, for the GLT 20 $\times$ 40, the cracks extend almost over two-thirds of the cross-sectional width. In summary, the larger the initial MC, the larger the potential $d_{\textrm{c}}^{\textrm{max}}$, which highlights why an initial MC below 12 % after production is preferred. Although more drying energy and time are required to ensure these moisture levels, a significantly lower loss in the load-bearing capacity can be expected.

For all cross sections, exponential crack growth can be seen, especially for the GLT 20 $\times$ 40. However, $d_{\textrm{c}}^{\textrm{max}}$ may be underestimated, if the initial MC is reduced from 11.9 % to as low as 8.2 % and the initial MC is reduced from 10.0 % to as low as 6.3 %, since the corresponding data points are assessed as outliers in the curve fitting process. Additionally, for the GLT 20 $\times$ 40, it can be noticed that the cracked width in case of decreasing the initial MC from 15.3 % to 6.3 % over 22.5 d is probably underestimated, since moisture flux is still present afterward. Besides, for the ST 6 $\times$ 8 and ST 14 $\times$ 28, $d_{\textrm{c}}^{\textrm{max}}$ could be larger in case of an initial MC of 15.3 %, and larger sudden reductions of the RH ($\Delta u_{\textrm{equ}}>$ 12 % for the ST 6 $\times$ 8 and $\Delta u_{\textrm{equ}}>$ 8 % for the ST 14 $\times$ 28), as the additional courses shown in Fig. 13 (dotted curves) indicate that $d_{\textrm{c}}^{\textrm{max}}$ varies under these conditions.

**Table 5:** Overview of the resulting maximum total crack depth $d_{\textrm{c}}^{\textrm{max}}$ given in relation to the total width of the cross section decreasing the initial moisture content (MC) from 15.3 % to 8.2 % over 30 d, to 7.3 % over 30 d and to 6.3 % over 22.5 d, respectively.

Initial MC (%)	Maximum total crack depth $d_{\textrm{c}}^{\textrm{max}}$ (%)								
	Decreased to 8.2 MC (%) (30 d)			Decreased to 7.2 MC (%) (30 d)			Decreased to 6.3 MC (%) (22.5 d)		
	ST 6$\times$8	ST 14$\times$28	GLT 20$\times$40	ST 6$\times$8	ST 14$\times$28	GLT 20$\times$40	ST 6$\times$8	ST 14$\times$28	GLT 20$\times$40
10.0	0.3	0.0	0.0	1.2	1.9	0.0	3.0	8.9	12.2
11.9	0.3	8.4	5.4	2.2	16.9	36.4	7.3	25.9	54.2
15.3	27.7	34.3	62.2	39.2	37.9	66.4	48.2	40.4	64.8

## Conclusion and outlook

In this paper, the cracking behavior of three cross sections for different immediate reductions of RH and initial MCs was investigated numerically. Based on this, a relation between the maximum total crack depth $d_{\textrm{c}}^{\textrm{max}}$, which is defined as the sum of the deepest cracks at both the left and the right edge of a cross section, and the moisture gradient between two points was studied. With the simulation results, the prediction of $d_{\textrm{c}}^{\textrm{max}}$ in indoor climate conditions was enabled and simplified in a further step. It was assumed that wooden beams are installed under ideal conditions, where the MC in timber cross sections is kept constant during production until the beginning of operation. In addition, the influence of linear RH reductions over time on crack behavior was examined. To simulate the crack formation, a multi-Fickian transport model (Eitelberger et al. 2011; Fortino et al. 2013, 2019; Frandsen et al. 2007a; Konopka and Kaliske 2018; Krabbenhøft and Damkilde 2004) was used to identify the moisture field in a first step. Next, the time-dependent moisture distribution induced stresses leading to crack initiation and propagation were determined by XFEM simulations. In course of this, a multisurface failure criterion (Lukacevic et al. 2017), defining the failure behavior, and a multiscale material model (Hofstetter et al. 2005), describing the moisture-dependent stiffness tensor entries, were used. The main conclusions are summarized as follows:

- XFEM allows the determination of maximum crack depths without a significant influence of the mesh size.
- Crack initiation was evaluated based on elastic stress fields. For this reason, the obtained crack depths are to be considered as an upper limit and, therefore, as a conservative prediction.
- Moisture gradients could be meaningfully correlated to $d_{\textrm{c}}^{\textrm{max}}$ in indoor climate conditions. These relations change depending on the cross section’s size and initial MC.
- In general, larger cross sections exhibit larger moisture gradients, and therefore, cracks are initiated earlier and crack growth tends to last longer.
- The initial MC significantly influences $d_{\textrm{c}}^{\textrm{max}}$. The quantification of this well-known phenomenon could become important in practical applications.
- For small cross sections, HVAC systems could be used to lower the RH continuously over a time span to reduce moisture gradients and, thus, decrease the crack depth significantly. For larger cross sections, a lower initial MC is recommended to avoid moisture induced cracking.

At this point, it should be emphasized that the main conclusions are only based on simulations performed on three different cross sections. To confirm the findings, additional simulations or experiments with a larger variety of cross sections are necessary. Based on these, a sufficiently accurate $d_{\textrm{c}}^{\textrm{max}}$ determination seems possible for installations, where the cross sections are protected from environmental influences after production to maintain equilibrium MC levels until the start of operation.

For the ST 14 $\times$ 28, deep vertical cracks occurred, but not for the ST 6 $\times$ 8, which is related to the height-to-width ratio as additional simulations confirmed. Further investigations to discover all aspects of vertical crack development could be a further step.

For future work, the cracking behavior of other engineered wood products and wood composite structures in indoor climate conditions could be investigated, as, e.g., timber-concrete composite structures. Lukacevic et al. (2021) showed that higher MCs in the CLT plate could be expected due to the bleeding of fresh concrete, and combined with a low surrounding RH, a considerable chance of moisture-induced cracking is given.

In addition, based on the established relation between moisture gradients and $d_{\textrm{c}}^{\textrm{max}}$, an approximate estimation crack depth development of timber cross sections subjected to outdoor climate conditions seems possible.

Furthermore, viscoelastic, plastic and the mechano-sorptive (reversible and irreversible) strains could be implemented to enable a more realistic stress determination, leading to a reduced stress level compared to a simulation based only on linear elastic material behavior.

With the presented results, a basis to predict crack depths in indoor climate conditions after an ideally conditioned installation, where the MC is kept constant during production until the beginning of operation, was developed, which could be used for the development of software tools applicable for engineers.

## Supplementary Information

Below is the link to the electronic supplementary material.

Supplementary file 1 (pdf 309 KB)

## Data Availability

On request.

## Abbreviations

$(\Delta u / \Delta x)_{\mathrm {P_1P_2}}$: the moisture gradient, which is defined as the difference in MC between two points P$_1$ and P$_2$ divided by their distance, where P$_1$ and P$_2$ are based on positions of finite element nodes (%/mm)
$\Delta u_{\textrm{equ}}$: difference in the equilibrium moisture content (%)
$\overline{\textrm{h}}_{\textrm{b}}$: average enthalpy of bound water (J kg$^{-1}$)
$\rho _{\textrm{d}}$: density of dry wood (kg m$^{-3}$ )
$d_{\textrm{c}}^{\textrm{max}}$: sum of the deepest cracks at both the left and the right edge, defined as the maximum total crack depth (mm)
$\dot{c}_{bv}$: sorption rate of bound water and water vapor (kg m$^{-3}$ s$^{-1}$)
$\phi _T$: boundary heat flux (W m$^{-2}$)
$\phi _v$: boundary flux of water vapor (kg m$^{-2}$ s$^{-1}$)
$c_{b}$: bound water concentration (kg m$^{-3}$)
$c_{v}$: water vapor concentration (kg m$^{-3}$)
$k_{T}$: heat transfer coefficient (W m$^{-2}$ K$^{-1}$)
$k_{{c}_v}$: mass transfer coefficient for water vapor (m s$^{-1}$)
*T*: temperature (K)
**c**$_{\textrm{p}_{\textrm{s}}}$: specific isobaric heat capacity of solid material matrix (J kg$^{-1}$ K$^{-1}$)
**D**$_{\mathbf {\textrm{b}}}$: diffusion tensor of bound water (m$^{2}$ s$^{-1}$)
**D**$_{\mathbf {\textrm{V}}}$: diffusion tensor of water vapor (m$^{2}$ s$^{-1}$)
**K**: conductivity tensor (W m$$^{-1}$$ K$$^{-1}$$)
f$_{\textrm{lum}}$: volume fraction of the lumen (-)
FSP: fiber saturation point
h$_{\textrm{b}}$: enthalpy of bound vapor (J kg$^{-1}$)
h$_{\textrm{v}}$: enthalpy of water vapor (J kg$^{-1}$)
MC: moisture content (%)
RH: relative humidity (%)

## Declarations

### Code availability

On request.
