# Supplementary material for: Prediction of moisture-induced cracks in wooden cross sections using finite element simulations
Source: Wood Sci Technol. 2023 Apr 27;57(3):671–701. doi: 10.1007/s00226-023-01469-3 (PMC10185615; doi:10.1007/s00226-023-01469-3)
Supplement: Supplementary file 1 — (pdf 309 KB) [file 226_2023_1469_MOESM1_ESM.pdf]

Prediction of moisture-induced cracks in wooden cross sections using finite element simulations

Wood Science and Technology

Florian Brandstätter[1*], Maximilian Autengruber[1], Markus Lukacevic[1] and Josef Füssl[1]

[1*]TU Wien, Institute for Mechanics of Materials and Structures, Karlsplatz 13, Vienna, 1040, Austria.

*Corresponding author E-mail: florian.brandstaetter@tuwien.ac.at;

1 Material properties and constitutive equations for the multi-Fickian model

To calculate the thermodynamic properties for specific isobaric heat capacities c_p and enthalpies h the reference state for the constitutive equations is set to 273.15 K and 101 325 Pa.

Table 1 Material parameters as well as constitutive equations for determination of the moisture and heat transport.

Property	Value/Constitutive equation	Ref.
Dry density	$\rho_d = 420\,\mathrm{kg\,m^{-3}}$	
Moisture content	$u = \frac{c_b}{\rho_d}$	
Bound water diffusion tensor	$\boldsymbol{D_b} = \boldsymbol{D_0}\,\exp\left(\frac{-E_b}{R\,T}\right)$	(Frandsen, 2007)
Activation energy of bound water	$E_b = 38500 - 29000\,u$	(Siau, 1984)
Universal gas constant	$R = 8.314\,\mathrm{J\,mol^{-1}\,K^{-1}}$	
Water vapor diffusion tensor	$\boldsymbol{D_v} = \boldsymbol{\xi}\left(2.31\cdot 10^{-5}\,\frac{p_{atm}}{p_{atm}+p_{v_{air}}}\left(\frac{T}{273}\right)^{1.81}\right)$	(Schirmer, 1938; Frandsen et al, 2007a; Krabbenhøft and Damkilde, 2004)
Water vapor pressure	$p_{v_{air}} = c_v\,\frac{R\,T}{M_{H_2O}}$	
Atmospheric air pressure	$p_{atm} = 101\,325\,\mathrm{Pa}$	
Molar mass of water	$M_{H_2O} = 18.015\,\mathrm{g\,mol^{-1}}$	
Moist density of wood	$\rho_{moist} = \rho_d\,\frac{1+u}{1+0.84\,c_b}$	(Kollmann, 1951)
Volume proportion of the cell lumen	$f_{lum} = 1 - \frac{\rho_{moist}}{\rho_{cwm}}$	
Density of the pure cell wall material	$\rho_{cwm} = 1530\,\mathrm{kg\,m^{-3}}$	(Siau, 1984; Eitelberger et al, 2011)
Conduction tensor	$\boldsymbol{K} = \boldsymbol{K_0}\,(0.142 + 0.46\,u)$	(Perré and Turner, 1999)
Heat capacity of the cell wall material	$c_{p_s} = -0.60453 + 0.006714\,T$	(Yang, 2000)
Enthalpy of water vapor	$h_v = 2060.5 + 1.3798\,T + 0.84808\cdot 10^{-4}\,T^2$	(Eitelberger, 2011)
Specific enthalpy of bound water	$h_b = 4.185\,(T - 273.15\,\mathrm{K}) - 1146.4\,\exp(-14.48\,u)$	(Skaar, 1988; Eitelberger, 2011)
Average enthalpy of bound water	$\overline{h}_b = -1143.1 + 4.185\,T - \frac{79.172\,\rho_d\,(1-\exp(-14.48\,u))}{c_b}$	(Stanish et al, 1986; Turner, 1996; Eitelberger, 2011)

Table 2 Diagonal components of the material parameter tensors

Parameter	Component			Ref.
	L	R	T	
$\boldsymbol{D_0}$	$2.5 \cdot 7 \cdot 10^{-6}$	$7 \cdot 10^{-6}$	$7 \cdot 10^{-6}$	(Fortino et al, 2013)
$\boldsymbol{\xi}$	0.9	0.11	0.11	(Dvinskikh et al, 2011)
$\boldsymbol{K_0}$	2	1	1	(Perre et al, 1993)

Table 3 Expansion coefficients α for temperature (Weatherwax and Stamm, 1956) and β for moisture (Gloimüller et al, 2012)

	α [1/K]	β [%/%]
L	3.15 E-6	0.015
R	23.8 E-6	0.19
T	32.3 E-6	0.36

2 Bound water and water vapor interaction

To describe the interaction between bound water and water vapor the sorption rate is required, which can be determined using the model of Krabbenhøft and Damkilde (2004); Frandsen et al (2007b); Fortino et al (2013), as described in Autengruber et al (2020, 2021). A sorption isotherm defines the connection between a particular equilibrium MC and a specific RH where the shape factors $f_{1,a} = 1.804, f_{2,a} = 13.63, f_{3,a} = -12.12$ for adsorption and $f_{1,d} = 1,886, f_{2,d} = 7.884, f_{3,d} = -6.526$ for desorption characterized the isotherm. The coefficients defining the hysteresis are $d_1 = -1.3$ and $d_2 = 0.88$, as shown in Frandsen et al (2007b). The moisture-dependent reaction function H_{bv} is described by the parameters $C_{bv,1} = 3.8 \cdot 10^{-4}\,\mathrm{s}^{-1}, C_{bv,3} = 80$ and $C_{bv,4} = 5.94 \cdot 10^{-7}\,\mathrm{s}^{-1}$, as obtained in Dvinskikh et al (2011) as well as $C_{bv,2} = c_{21} \exp\left(c_{22}\,\mathrm{RH}\right) + c_{23} \exp\left(c_{24}\,\mathrm{RH}\right)$, where $c_{21} = 3.58, c_{22} = 2.21, c_{23} = 1.59 \cdot 10^{-3}$ and $c_{24} = 14.98$, as shown in Dvinskikh et al (2011).

3 Elasticity tensor of spruce

Table 4 Tensor components C_{iiii} [MPa] for spruce elasticity at 293.15 K with a dry density of 420 kg m^{-3} (Hofstetter et al, 2005).

MC [%]	C_{LLLL}	C_{RRRR}	C_{TTTT}	C_{LLRR}	C_{RRTT}	C_{TTLL}	C_{LRLR}	C_{LTLT}	C_{RTRT}
3	13981.39	1127.15	755.30	343.76	272.01	520.26	396.68	389.40	53.70
4	13824.31	1094.04	733.20	337.33	266.94	504.70	385.64	378.57	52.23
5	13669.80	1061.40	711.42	330.88	261.87	489.33	374.67	367.80	50.79
6	13517.80	1029.22	689.96	324.41	256.78	474.16	363.78	357.11	49.37
7	13368.23	997.51	668.82	317.92	251.67	459.21	352.97	346.49	47.97
8	13221.06	966.26	647.98	311.40	246.55	444.47	342.25	335.97	46.60
9	13076.22	935.48	627.46	304.86	241.40	429.95	331.63	325.55	45.24
10	12933.66	905.17	607.25	298.29	236.23	415.66	321.12	315.23	43.91
11	12793.34	875.33	587.34	291.68	231.03	401.60	310.72	305.02	42.59
12	12655.21	845.95	567.74	285.04	225.80	387.77	300.45	294.94	41.29
13	12519.23	817.05	548.45	278.37	220.55	374.19	290.31	284.99	40.00
14	12385.35	788.61	529.47	271.66	215.27	360.84	280.32	275.18	38.73
15	12253.54	760.65	510.79	264.92	209.95	347.74	270.47	265.51	37.47
16	12123.75	733.16	492.42	258.14	204.61	334.88	260.78	256.00	36.23
17	11995.96	706.15	474.35	251.33	199.24	322.26	251.25	246.64	35.01
18	11870.13	679.60	456.59	244.48	193.84	309.90	241.89	237.46	33.79
19	11746.22	653.53	439.14	237.61	188.41	297.77	232.71	228.44	32.60
20	11624.20	627.93	421.99	230.71	182.96	285.89	223.70	219.60	31.41
21	11504.05	602.80	405.15	223.78	177.49	274.26	214.88	210.94	30.24
22	11385.74	578.14	388.62	216.82	171.99	262.86	206.25	202.47	29.08
23	11269.23	553.95	372.39	209.85	166.48	251.71	197.81	194.18	27.94
24	11154.50	530.22	356.46	202.86	160.95	240.79	189.56	186.09	26.81
25	11041.52	506.95	340.83	195.86	155.41	230.11	181.51	178.18	25.70
26	10930.28	484.14	325.51	188.86	149.86	219.66	173.66	170.47	24.60
27	10820.74	461.80	310.49	181.85	144.30	209.44	166.00	162.96	23.52
28	10712.87	439.90	295.77	174.84	138.75	199.44	158.55	155.64	22.45
29	10606.66	418.46	281.35	167.84	133.20	189.67	151.29	148.52	21.40
30	10502.09	397.46	267.22	160.84	127.65	180.11	144.23	141.59	20.36

4 Tsai-Wu parameters

Table 5 Tsai-Wu tensor components and failure type for all surfaces $f_{\mathrm{i}}^{\mathrm{cw}}$ (Lukacevic et al, 2017).

Surface	Type	$a_{LL,\mathrm{i}}$	$a_{RR,\mathrm{i}}$	$a_{TT,\mathrm{i}}$	$b_{LLLL,\mathrm{i}}$	$b_{RRRR,\mathrm{i}}$	$b_{TTTT,\mathrm{i}}$	$b_{RRTT,\mathrm{i}}$	$b_{LRLR,\mathrm{i}}$	$b_{RTRT,\mathrm{i}}$	$b_{TLTL,\mathrm{i}}$
1	Crack	-0.00414	0.01173	0.47073	0.00008	0.00713	0.00559	0.00048	0.01235	0.01563	0.00826
2	Crack	-0.00370	0.12170	0.34478	0.00008	0.00405	0.00541	0.00138	0.01235	0.01563	0.00826
3	Crack	-0.00240	0.11788	-0.25892	0.00007	0.02081	0.00357	-0.00231	0.01235	0.01563	0.00826
4	Plastic	-0.00357	0.04885	-0.34852	0.00013	0.00890	0.00299	0.00002	0.01235	0.01563	0.00826
5	Plastic	0.00029	-0.01561	-0.18639	0.00011	0.01591	0.00517	0.00024	0.01235	0.01563	0.00826
6	Plastic	-0.00876	-0.03995	0.31830	0.00020	0.01897	0.00001	0.00000	0.01235	0.01563	0.00826
7	Crack	-0.02167	-0.00909	-0.10714	0.00008	0.00909	0.03571	0.00000	0.01235	0.01563	0.00826
8	Plastic	0.01452	-0.03409	0.03333	0.00006	0.01136	0.03333	0.00000	0.01235	0.01563	0.00826

5 Reducing relative humidity linearly over time

Table 6 Overview of the resulting maxima of d_c^{max}, $(\Delta u/\Delta x)_{BC}$ and $(\Delta u/\Delta x)_{B_{15}B_{25}}$ when the initial RH of 65 % (15.3 % initial MC) is reduced to 25.2 % (7.3 % MC) linearly over times.

	ST 6 × 8				ST 14 × 28				GLT 20 × 40			
	0 d	5 d	10 d	25 d	0 d	15 d	30 d	45 d	0 d	15 d	30 d	45 d
max. d_c^{max} [mm]	25	14	7	2	56	52	48	44	135	131	126	116
max. $(\Delta u/\Delta x)_{BC}$ [%/mm]	0.216	0.205	0.136	0.093	0.103	0.101	0.097	0.089	0.074	0.073	0.072	0.071
max. $(\Delta u/\Delta x)_{B_{15}B_{25}}$ [%/mm]	0.155	0.144	0.092	0.063	0.184	0.167	0.145	0.127	0.185	0.168	0.146	0.130

References

Autengruber M, Lukacevic M, Füssl J (2020) Finite-element-based moisture transport model for wood including free water above the fiber saturation point. International Journal of Heat and Mass Transfer 161:120,228. https://doi.org/https://doi.org/10.1016/j.ijheatmasstransfer.2020.120228, URL http://www.sciencedirect.com/science/article/pii/S0017931020331641

Autengruber M, Lukacevic M, Gröstlinger C, et al (2021) Finite-element-based prediction of moisture-induced crack patterns for cross sections of solid wood and glued laminated timber exposed to a realistic climate condition. Construction and Building Materials 271:121,775. https://doi.org/https://doi.org/10.1016/j.conbuildmat.2020.121775, URL https://www.sciencedirect.com/science/article/pii/S095006182033779X

Dvinskikh SV, Henriksson M, Mendicino AL, et al (2011) Nmr imaging study and multi-fickian numerical simulation of moisture transfer in norway spruce samples. Engineering Structures 33:3079–3086. URL http://www.sciencedirect.com/science/article/pii/S0141029611001726

Eitelberger J (2011) A multiscale material description for wood below the fiber saturation point with particular emphasis on wood-water interactions. PhD thesis, Vienna University of Technology

Eitelberger J, Hofstetter K, Dvinskikh S (2011) A multi-scale approach for simulation of transient moisture transport processes in wood below the fiber saturation point. Composites Science and Technology 71(15):1727–1738. URL http://www.sciencedirect.com/science/article/pii/S0266353811002946

Fortino S, Genoese A, Genoese A, et al (2013) Numerical modelling of the hygro-thermal response of timber bridges during their service life: A monitoring case-study. Construction and Building Materials 47:1225–1234. URL http://www.sciencedirect.com/science/article/pii/S0950061813005278

Frandsen HL (2007) Selected constitutive models for simulating the hygromechanical response of wood. PhD thesis, Aalborg University

Frandsen HL, Damkilde L, Svensson S (2007a) A revised multi-fickian moisture transport model to describe non-fickian effects in wood. Holzforschung 61:563–572. URL http://www.degruyter.com/view/j/hfsg.2007.61.issue-5/hf.2007.085/hf.2007.085.xml

Frandsen HL, Svensson S, Damkilde L (2007b) A hysteresis model suitable for numerical simulation of moisture content in wood. Holzforschung 61:175–181. URL http://www.degruyter.com/view/j/hfsg.2007.61.issue-2/hf.2007.031/hf.2007.031.xml

Gloimüller S, De Borst K, Bader T, et al (2012) Determination of the linear elastic stiffness and hygroexpansion of softwood by a multilayered unit cell using poromechanics. Interaction and multiscale mechanics 5(3):229–265

Hofstetter K, Hellmich C, Eberhardsteiner J (2005) Development and experimental validation of a continuum micromechanics model for the elasticity of wood. European Journal of Mechanics - A/Solids 24(6):1030–1053. URL http://www.sciencedirect.com/science/article/pii/S0997753805000963

Kollmann F (1951) Technologie des Holzes und der Holzwerkstoffe: 1. Band. Springer, Berlin, Heidelberg, URL https://books.google.at/books?id=T5GTBwAAQBAJ

Krabbenhøft K, Damkilde L (2004) A model for non-fickian moisture transfer in wood. Materials and Structures 37(9):615–622. URL http://dx.doi.org/10.1007/BF02483291

Lukacevic M, Lederer W, Füssl J (2017) A microstructure-based multisurface failure criterion for the description of brittle and ductile failure mechanisms of clear-wood. Engineering Fracture Mechanics 176:83–99. URL https://www.sciencedirect.com/science/article/pii/S0013794416307603

Perre P, Moser M, Martin M (1993) Advances in transport phenomena during convective drying with superheated steam and moist air. International Journal of Heat and Mass Transfer 36(11):2725–2746. URL http://www.sciencedirect.com/science/article/pii/001793109390093L

Perré P, Turner IW (1999) A 3-d version of transpore: a comprehensive heat and mass transfer computational model for simulating the drying of porous media. International Journal of Heat and Mass Transfer 42(24):4501–4521. URL http://www.sciencedirect.com/science/article/pii/S0017931099000988

Schirmer R (1938) Die Diffusionszahl von Wasserdampf-Luft-Gemischen und die Verdampfungsgeschwnidigkeit. VDI Beiheft Verfahrenstechnik 1938(6):170–177

Siau JF (1984) Transport Processes in Wood. Springer, Berlin, Heidelberg, https://doi.org/https://doi.org/10.1007/978-3-642-69213-0

Skaar C (1988) Wood-Water Relations. Springer-Verlag Berlin Heidelberg, https://doi.org/10.1007/978-3-642-73683-4

Stanish MA, Schajer GS, Kayihan F (1986) A mathematical model of drying for hygroscopic porous media. AIChE J 32(8):1301–1311. https://doi.org/10.1002/aic.690320808, URL https://doi.org/10.1002/aic.690320808

Turner IW (1996) A two-dimensional orthotropic model for simulating wood drying processes. Applied Mathematical Modelling 20(1):60–81. URL http://www.sciencedirect.com/science/article/pii/0307904X9500106T

Weatherwax RC, Stamm AJ (1956) The coefficients of thermal expansion of wood and wood products. Tech. Rep. 1487, US Forest Products Laboratory

Yang Q (2000) Study on the specific heat of wood by statistical mechanics. Journal of Forestry Research 11(4):265–268. URL https://doi.org/10.1007/BF02844975
